# Predicting Successful Weaning from Mechanical Ventilation by Reduction in Positive End-expiratory Pressure Level Using Machine Learning

**DOI:** 10.1371/journal.pdig.0000478

**Published:** 2024-03-27

**Authors:** Seyedmostafa Sheikhalishahi, Mathias Kaspar, Sarra Zaghdoudi, Julia Sander, Philipp Simon, Benjamin P. Geisler, Dorothea Lange, Ludwig Christian Hinske

**Affiliations:** 1 Digital Medicine, University Hospital of Augsburg, Augsburg, Germany; 2 Anesthesiology and Surgical Intensive Care Medicine, University Hospital of Augsburg, Augsburg, Germany; 3 Massachusetts General Hospital, Harvard Medical School, Boston, Massachusetts, United States of America; 4 Department of Anesthesiology, LMU University Hospital, Munich, Germany; University of Washington, UNITED STATES

## Abstract

Weaning patients from mechanical ventilation (MV) is a critical and resource intensive process in the Intensive Care Unit (ICU) that impacts patient outcomes and healthcare expenses. Weaning methods vary widely among providers. Prolonged MV is associated with adverse events and higher healthcare expenses. Predicting weaning readiness is a non-trivial process in which the positive end-expiratory pressure (PEEP), a crucial component of MV, has potential to be indicative but has not yet been used as the target. We aimed to predict successful weaning from mechanical ventilation by targeting changes in the PEEP-level using a supervised machine learning model. This retrospective study included 12,153 mechanically ventilated patients from Medical Information Mart for Intensive Care (MIMIC-IV) and eICU collaborative research database (eICU-CRD). Two machine learning models (Extreme Gradient Boosting and Logistic Regression) were developed using a continuous PEEP reduction as target. The data is splitted into 80% as training set and 20% as test set. The model’s predictive performance was reported using 95% confidence interval (CI), based on evaluation metrics such as area under the receiver operating characteristic (AUROC), area under the precision-recall curve (AUPRC), F1-Score, Recall, positive predictive value (PPV), and negative predictive value (NPV). The model’s descriptive performance was reported as the variable ranking using SHAP (SHapley Additive exPlanations) algorithm. The best model achieved an AUROC of 0.84 (95% CI 0.83–0.85) and an AUPRC of 0.69 (95% CI 0.67–0.70) in predicting successful weaning based on the PEEP reduction. The model demonstrated a Recall of 0.85 (95% CI 0.84–0.86), F1-score of 0.86 (95% CI 0.85–0.87), PPV of 0.87 (95% CI 0.86–0.88), and NPV of 0.64 (95% CI 0.63–0.66). Most of the variables that SHAP algorithm ranked to be important correspond with clinical intuition, such as duration of MV, oxygen saturation (SaO_2_), PEEP, and Glasgow Coma Score (GCS) components. This study demonstrates the potential application of machine learning in predicting successful weaning from MV based on continuous PEEP reduction. The model’s high PPV and moderate NPV suggest that it could be a useful tool to assist clinicians in making decisions regarding ventilator management.

## Author summary

The weaning of patients from mechanical ventilation (MV) in the intensive care unit is crucial for patient outcomes and healthcare costs. This retrospective study explores the application of machine learning to predict successful weaning from MV, focusing on the positive end-expiratory pressure (PEEP), a key component of MV, as a potential predictor for successful weaning. Analyzing data of 12,153 patients from eICU-CRD and MIMIC-IV, we developed models using Extreme Gradient Boosting (XGBoost) and Logistic Regression (LR), targeting PEEP reduction. Our best model showed an AUROC of 0.84, an AUPRC of 0.69, and practical utility in predicting weaning success, validated by metrics such as Recall, F1-score, PPV, and NPV. Important variables identified by the SHAP algorithm, such as duration of MV and oxygen saturation aligned with clinical intuition. These findings highlight the potential of machine learning in enhancing ICU ventilator management, contributing to improved patient care and resource utilization efficiency.

**Summary table**

What was already known on the topic?

- Weaning from mechanical ventilation is a complex process influencing the patient outcome
- Positive end-expiratory pressure (PEEP) to predict weaning is evident, but was not used before

What did this study add to our knowledge?

- We showed the viability of using a new method targeting the PEEP measurement to predict weaning
- Prediction results varies among different datasets

## Introduction

Globally, between 50–70% of patients in the intensive care unit (ICU) are in need of mechanical ventilation (MV) during their ICU stay [1–5]. While non-invasive MV utilizes masks or helmets that cover the patient’s mouth and/or nose to support patients’ breathing efforts [6], invasive MV involves endotracheal intubation. The ventilator then delivers oxygen into the patient’s lungs via positive pressure, establishing an artificial breathing mechanism. To prevent the lung’s alveoli from collapsing during this process, a small amount of pressure is maintained at the end of each breathing cycle, called positive end-expiratory pressure (PEEP). It is critical for clinicians to determine the right time to start the process of liberating the patient from MV, which is called weaning [7]. Failure to wean patients from MV is associated with a longer ICU stay and adverse events including an increased mortality [8,9]. In 2005, an International Consensus Conference in Budapest provided basic guidelines for the complex process of weaning [10]. Many studies have examined the key clinical parameters that should be considered when deciding on the appropriate timing for weaning, as summarized by Thille et. al [8]. Several studies have used such parameters to examine the ability of machine learning (ML) to predict weaning, based on different cohorts and from different perspectives.

Most of the related work defines weaning as a binary classification of having been fully weaned from invasive MV in the form of an extubation [11–16]. A smaller number of authors describe algorithms that handle weaning as a multistage process, e.g., in terms of predicting the change from control to support mode first to subsequently predict extubation [17–19]. The latter approach better captures the complexity of the entire weaning process, which should be interpreted as a continuous process rather than a single event, underlining the intricacy of predicting the weaning from MV.

In this context, Otaguro et al. [11] carried out a study using a single electronic health records data for patients intubated after respiratory failure receiving MV. They employed Random Forest, Extreme Gradient Boosting (XGBoost), and light gradient boosting machine (LightGBM) algorithms to predict extubation without intubation within the next 72 hours. Similarly, Lin et al. [12] focused on predicting successful weaning in patients requiring prolonged MV who were admitted to the respiratory care center. They defined successful weaning as being liberated from MV for five consecutive days. The full weaning algorithm described by Liao et. al. [14] uses XGBoost, they developed an Artificial Intelligence prediction dashboard to predict successful weaning from MV. Similarly, Jia et. al. [15] predicts a full weaning using a Convolutional Neural Network based on Medical Information Mart for Intensive Care—III (MIMIC-III) data. Liu et. al. [16] defined weaning as 48h without ventilation or death and they used MIMIC-IV data as training set and test set and eICU collaborative research database (eICU-CRD) data for external validation to predict a full weaning. One of the latest studies by Zhao et al. [20] used data from MIMIC-IV to train and evaluate a CatBoost algorithm to predict extubation failure as the need for re-intubation or death within 48-hour intervals following the planned extubation. Strodthoff et al. [17] used data from both MIMIC-III and eICU-CRD with random forest algorithms, individual neural networks, and a multi-tasking network to predict relevant ventilation parameters instead of a binary outcome (PaO_2_, partial arterial carbon dioxide pressure, and respiratory system compliance) 30 minutes into the future. There is only little work done on multi-stage weaning. In this context, Liu et. al. [18] described a two-stage weaning prediction algorithm based on XGBoost and other algorithms; first they successfully predict the switch of the ventilator from control to support mode, and from this stage they predict successful weaning. Similarly, Cheng et. al [19] define weaning by a mode downshift, e.g., from full-support to partial-support mode.

The PEEP value is one of the parameters of MV which captures several aspects of the weaning process at once: First, as increasing PEEP-levels are used to enhance oxygenation [21], the level of PEEP can be a surrogate parameter for the lungs oxygenating properties (where an increasing PEEP suggest worsening, a decreasing PEEP improving oxygenation). Second, as higher PEEP-levels are applied to prevent derecruitment and atelectasis (“collapsed” lung areas), thus reducing ventilation-perfusion mismatch [22], patients tolerating a lower PEEP-level can be suspected to have sufficient ventilation. Finally, yet importantly, PEEP can increase lung compliance and decrease work of breathing [23]. In this case, a reduction in PEEP-levels can be perceived as a recovery of breathing mechanics. It’s worth noting that the International Consensus Conference in 2005 ruled a PEEP-level of 8 cmH2O or less as an indicator for “minimal ventilator dependency”, making it one of the required criteria for weaning from mechanical ventilation.

For everyday clinical practice this means that successful weaning is basically always preceded by a reduction in PEEP, which however has not yet been used to predict successful weaning. In this study, we aimed to predict successful weaning from mechanical ventilation using a supervised machine learning model by targeting changes in PEEP-levels, defining a continuous PEEP reduction without de novo increase as weaning success.

## Methods

### Patient data selection

**Fig 1:**
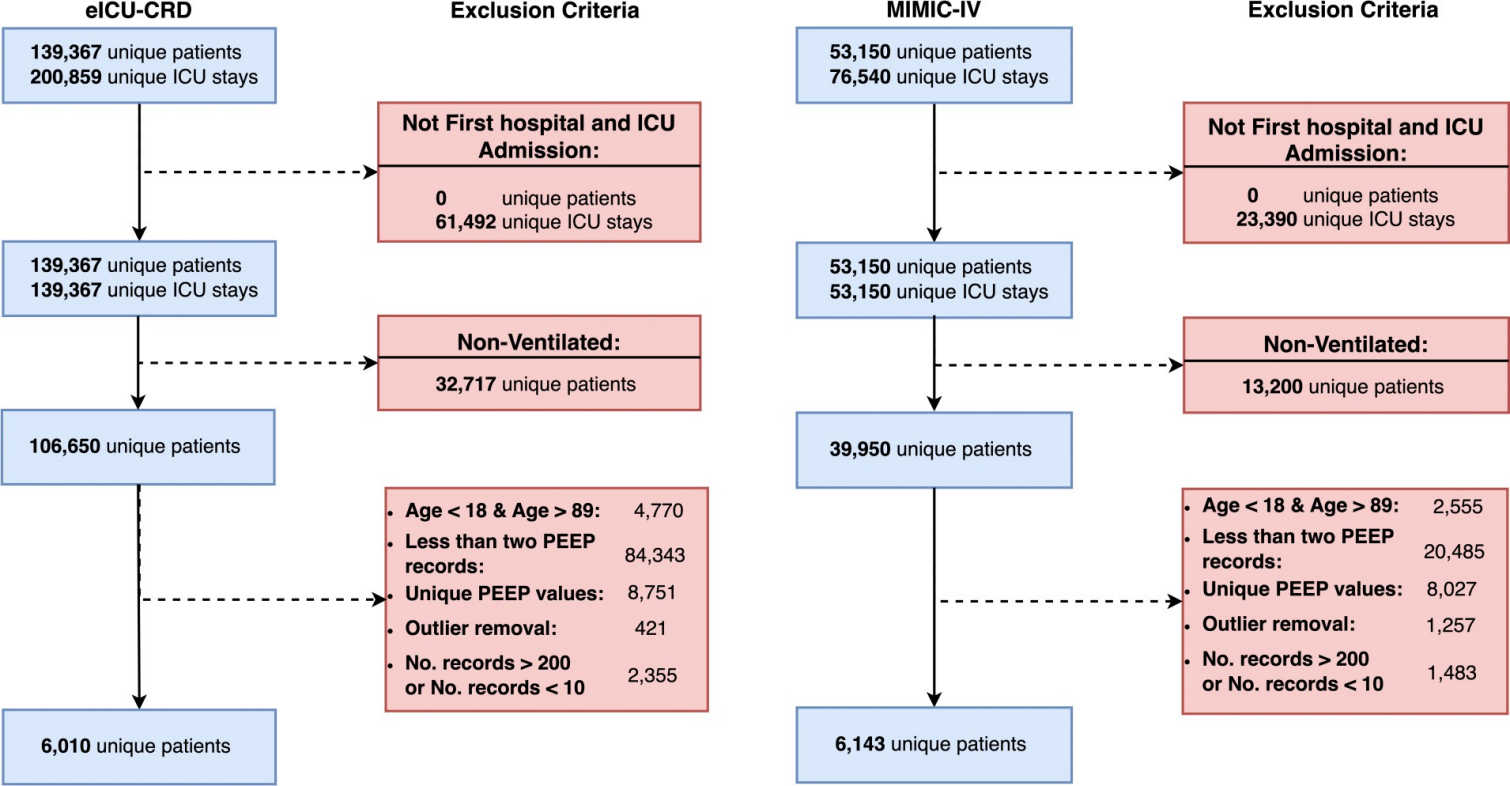
Study flow diagram. This graph describes the stepwise application of exclusion criteria to the entire dataset (top-most box) towards the final study selection (bottom-most box) for eICU collaborative research database (eICU-CRD) on the left panel and Medical Information Mart for Intensive Care (MIMIC-IV) on the right panel. The number of patients is equal to the number of admissions after the application of the first criteria.

This study utilizes data from the MIMIC-IV v1.0 [24] and eICU-CRD [25] datasets. Both are US-based critical care databases with 76,540 and 200,859 ICU admissions, respectively. In this study, we included the first ICU admission and hospital admissions of patients from different ICUs, between 18 and 89 years of age, and on MV with at least two recorded PEEP measurements. Furthermore, we included patients with more than 10 and less than 200 observations to ensure a careful equilibrium and capture an adequate amount of data for insightful analysis while mitigating the biases associated with excessively small or large numbers of observations. Fig 1 illustrates the exclusion criteria applied to both datasets.

### Outcome definition

**Fig 2:**
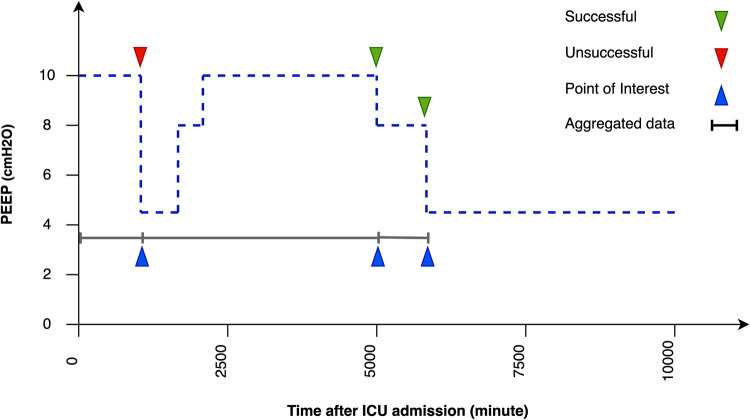
Problem definition using the PEEP value. Illustration of the problem statement with progression of a single patient’s PEEP-level during a stay in the ICU. In this regard, the event is the time at which the PEEP-level is decreased. Any PEEP-level decrease that is followed by an increase to at least the same level as before refers to a failed weaning (red triangle). A PEEP-level decrease without further increase to a previous level until the end of the ICU stay refers to a successful weaning (green triangle). Hence, in this figure there are one failed weaning and two successful weaning attempts.

This retrospective study aims to answer the following question: Is it the right time to decrease the PEEP-level as a crucial step in the weaning process? We have translated this question into a binary classification problem using data between the last weaning attempt or ICU admission and the current weaning attempt (event) to predict whether a PEEP-level decrease would be successful at the moment, as illustrated in Fig 2. Weaning is considered successful if PEEP decreases without a subsequent increase to a previous level until the end of the ICU stay, and failed if PEEP increases to at least the same level as before after a decrease before discharge.

### Variable selection and feature definition

We compiled 51 clinical variables identified by clinicians as relevant to predicting successful weaning, which have also been used in the literature and were available in both data sets (see S1 Appendix). Most variables have missing data that are largely comparable between datasets. Exceptions are some variables with higher missing rate in MIMIC (albumin +23%, SaO_2_ +23%) and in eICU-CRD (base excess +26%, GCS +31%, INR +23%, lactate +35%, MAP +26%, PIP +46%, PT +26%, PTT +38%). Missing values were forward-imputed. The remaining missing values were filled using a multi-variate imputer. For each of these variables, the mean, minimum, maximum, and standard deviation (SD) were calculated, constituting the 188 numeric features used in the ML models. Observation windows were the period from ICU admission to the current event or from the last event to the current event.

### Model development

We utilized two machine learning models of logistic regression as the baseline and XGBoost, as well as SHAP to provide an interpretation of the XGBoost output. Logistic regression (LR) employs a given set of independent variables to predict the output of a categorical dependent variable [26]. XGBoost uses an ensemble method in which different models are created sequentially. Thereby, a new model is created to reduce the errors of the previous model [27]. LR was chosen for its simplicity and interpretability in terms of a broad application, XGBoost for its accuracy, performance, and handling of complex, high-dimensional, and imbalanced data [28–30].

SHAP [31] is based on a classic method for distributing total gains in a cooperative game among the cooperating participants, which is adopted to approximate the contribution of each variable to the prediction in machine learning models. The method approximates the Shapley values of a given prediction by examining the impact of removing the given variable under all possible combinations of the presence or absence of the other remaining variables. SHAP is a unique method as it possesses several desirable properties including local accuracy, global consistency, and a clear probabilistic interpretation, making it an effective tool for interpretable machine learning and fairness evaluation.

### Model training and evaluation

**Fig 3:**
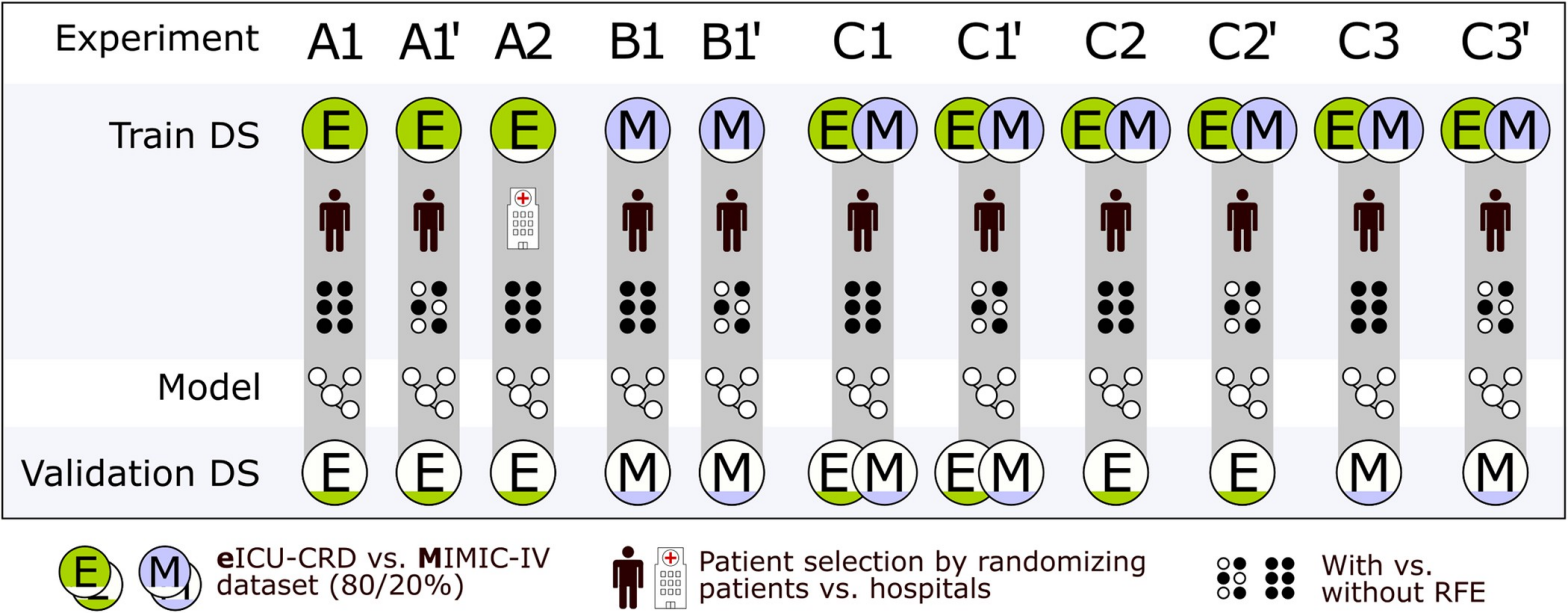
Experimental setup. We developed 11 models on the eICU-CRD (A) and MIMIC-IV (B) datasets (DS), solely and in combination (C), with and without recursive feature elimination (RFE).

In all experiments, patients including all of their data were randomly selected into train or test data sets, using a random train-test split method. The only exception is a single experiment using eICU-CRD data, where we did the same train-test split fraction on full hospital data. The model is trained on 80% of the data for each of the databases MIMIC-IV, eICU-CRD, and a combination of both. Each event and associated input were included separately from the patient in the training. The model is evaluated on the remaining 20% of the data. The hyperparameters are tuned manually based on trial and error. Each of these models was trained using the original set of 188 features. We also used the recursive feature elimination (RFE) algorithm to reduce the number of features while retaining the most relevant for the machine learning models. An overview of the experiments is illustrated in Fig 3. For A1, for example, a model is trained using LR and XGBoost on eICU-CRD data, a train-test split of 80/20% of the patients using all clinical variables and evaluated on eICU-CRD data. The only difference in A1’ is that we additionally applied RFE. Details are also described with the data in the results. Furthermore, we have done subgroup analyses related to age, sepsis, and lung function represented by the Horowitz index which are described in the S1 Appendix. The predictive performance is reported using 95% CI, based on evaluation metrics such as area under the receiver operating characteristic (AUROC), area under the precision-recall curve (AUPRC), F1-Score, Recall, positive predictive value (PPV), and negative predictive value (NPV). Result qualities are visualized using the receiver operating characteristic (ROC) curves. To visualize the reliability of the model, calibration curves are provided showing the frequency of the outcome versus the predicted probability. True/False-Positive/Negative metrics of all experiments are reported in the S1 Appendix. The 95% CI was computed via normal approximation. We compared the performance of different ML models using the CI and the performance metrics.

### Ethics statement

The use of the eICU-CRD [25] in research is not subject to institutional review board (IRB) approval. This exemption is due to the retrospective nature of the study design, the absence of direct patient intervention, and the secure data handling procedures. The re-identification risk associated with the dataset has been evaluated and certified as meeting safe harbor standards by an independent privacy expert, Privacert, based in Cambridge, MA (Health Insurance Portability and Accountability Act Certification no. 1031219–2). On the other hand, the data within MIMIC-IV v1.0 [24] has already undergone de-identification, and its use for research has been granted approval by both the institutional review boards of the Massachusetts Institute of Technology (No. 0403000206) and Beth Israel Deaconess Medical Center (2001-P-001699/14).

## Results

We included 12,153 patients in the study, 6,010 patients from eICU-CRD (38.4% female, age 62.3 years) and 6,143 patients from MIMIC-IV (42.2% female, age 62.9 years). Table 1 presents a patient characteristic of both datasets. All features used are described in the S1 Appendix.

**Table 1:** Patient characteristics. Basic socio-demographics and encounter parameters are provided for all included patients and divided by train and test dataset for eICU-CRD and MIMIC-IV.

Variables	eICU-CRD			MIMIC-IV		
	all (n = 6,010)	train (n = 4,808)	test (n = 1,202)	all (n = 6,131)	train (n = 4,904)	test (n = 1,227)
Age, mean (SD)	62.3 (15.1)	64 (16.2)	64 (14.8)	62.9 (15.4)	62.8 (15.5)	63.3 (15)
Female, n (%)	2,539 (42.2)	2,038 (42.3)	501 (41.7)	2,355 (38.4)	1,870 (38.1)	485 (39.6)
Height, mean (SD)	170.1 (10.6)	170.1 (10.6)	170.2 (10.6)	170.1 (9.3)	170 (9.3)	170 (9.1)
Weight, mean (SD)	86.5 (23.6)	86.3 (23.7)	87.5 (23.5)	84.9 (21.5)	84.9 (21.5)	84.8 (21.3)
Care unit, n (%)						
CVICU	427 (7.1)	345 (7.2)	82 (6.8)	1,629 (26.6)	1307 (26.7)	322 (26.2)
MICU	541 (9.0)	419 (8.7)	122 (10.1)	1,193 (19.5)	938 (19.1)	255 (20.8)
TSICU	650 (10.8)	512 (10.6)	138 (11.5)	1,767 (28.8)	1,420 (29.0)	347 (28.3)
Med-Surg ICU	3,164 (52.6)	2547 (53.0)	617 (51.3)	807 (13.2)	641 (13.1)	166 (13.5)
CCU	563 (9.4)	448 (9.3)	115 (9.6)	561 (9.2)	463 (9.4)	98 (8.0)
Neuro SICU	300 (5.0)	242 (5.0)	58 (4.8)	174 (2.8)	135 (2.8)	39 (3.2)
CTICU	209 (3.5)	169 (3.5)	40 (3.3)	n/a	n/a	n/a
CSICU	156 (2.6)	126 (2.6)	30 (2.5)	n/a	n/a	n/a
Ethnicity, n (%)						
White	4687 (77)	3748 (78)	939 (78)	5585 (65)	4442 (65)	1143 (65)
Black	406 (10)	488 (10)	111 (10)	714 (8)	573 (8)	141 (8)
Other	599 (7)	316 (7)	90 (7)	1650 (19)	1315 (19)	335 (19)
Hispanic	219 (4)	180 (3)	39 (3)	347 (4)	264 (4)	83 (5)
Asian	74 (1)	58 (1)	16 (1)	251 (3)	210 (3)	41 (2)
Native	25 (1)	18 (1)	7 (1)	33 (1)	23 (1)	10 (1)
MV duration [hour], mean (SD)	56.4 (57.2)	55.9 (56.1)	58.3 (61.3)	39.1 (38)	39.6 (38.8)	37.2 (34.4)
Heart rate, bpm (SD)	87.3 (15.5)	87.5 (15.6)	86.3 (15.3)	85.1 (14.7)	85 (14.6)	85.1 (15)
Mortality, n (%)	980 (16.3)	812 (16.9)	168 (14)	985 (16)	759 (15.5)	226 (18.4)

CSICU, cardiac surgery intensive care unit; CTICU, cardiothoracic intensive care unit; CVICU, cardiac vascular intensive care unit; CCU, coronary care unit; Med-Surg ICU, medical/surgical intensive care unit; MICU, medical intensive care unit; Neuro SICU, neurosurgical intensive care unit; TSICU, trauma surgical intensive care unit.

In eICU-CRD, we identified 20,162 events (35% positive), with a mean (SD) 3.35 (7.03) events per patient (1.45 (0.95) and 5.43 (9.87) for patients with at least one positive and negative event, respectively). In MIMIC-IV, we identified 10,700 events (70% positive), with a mean (SD) 1.75 (1.16) events per patient (1.41 (0.75) and 1.62 (0.96) for patients with at least one positive and negative event, respectively).

### eICU-CRD (A)

We carried out three experiments using the eICU-CRD dataset: A1) The full set of 188 features was used to train on a random selection of patients, regardless of hospital admission; A1’) RFE was applied to (A1) to reduce the features to 22 relevant features (20 clinical variables); A2) The same 188 features as in (A1) were used to train on full hospital data using 80% of the eICU-CRD hospitals (test on the remaining 20% of hospitals), to examine potential differences in treatment effects across hospitals.

**Fig 4:**
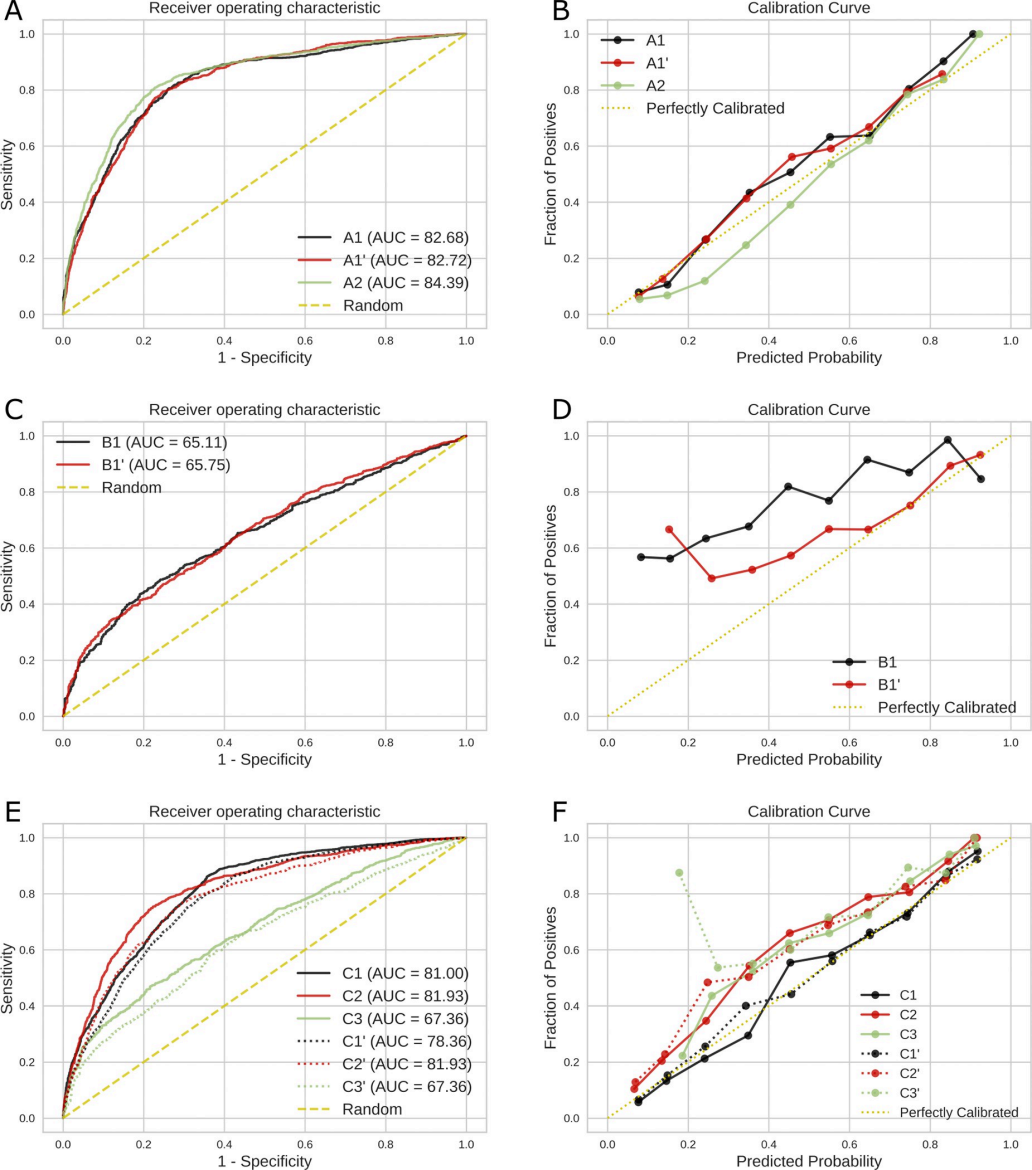
**AUROC (left panel) and Calibration curves (right panel) of XGBoost on eICU-CRD (A,B), on MIMIC-IV (C,D), and on the combined dataset (E,F).** Experiments: A1) Training and test set split based on patients using eICU-CRD, A1’) RFE applied to A1, A2) Similar to A1 with training and test set split based on full hospitals, B1) Similar to A1 using MIMIC-IV, B1’) RFE applied to B1, C1) Training and test on a combination of both datasets, C2) Training on a combination of both datasets and test on eICU-CRD (C2), Training on a combination of both datasets and test on MIMIC-IV (C3), and (C1’-C3’) RFE applied to C1, C2, C3.

Corresponding performance metrics are presented in Table 2. XGBoost showed statistically significant superiority over the LR method (baseline) in most experiments with a 95% CI, therefore, we limited the visual presentation in Fig 4A/4B to XGBoost. Performance metrics in experiments (A1) and (A1’) are comparable. In experiment (A2) compared to (A1) and (A1’), especially the F1-score, PPV, and AURUC are higher, reflected by the curve in Fig 4A, indicating improved predictive ability of patient sets selected entirely from individual hospitals as opposed to randomly selected from different hospitals. Fig 4B shows the calibration curve of XGBoost; in this context experiments (A1), (A1’), and (A2) are well calibrated. In the subgroup analysis, the models for age and sepsis had worse results, but the model with a Horowitz index ≥162 had a slightly better AUC compared to the main analysis.

**Table 2:** Model performance using the eICU-CRD dataset. Comparison of selected metrics for XGBoost and LR (baseline) on the defined experiments, see Fig 3. The best-performing metric values are represented in bold font.

Experiment	AUROC (95% CI)	AUPRC (95% CI)	Recall (95% CI)	F1-score (95% CI)	PPV (95% CI)	NPV (95% CI)
(A1) 80% training, 20% test, randomly selected ***patients***						
XGBoost	**0.82 (0.81–0.83)**	**0.72 (0.71–0.74)**	0.83 (0.82–0.84)	0.81 **(**0.80–0.83**)**	**0.80 (0.78–0.81)**	**0.60 (0.68–0.71)**
Logistic Regression	0.79 (0.78–0.80)	0.66 (0.64–0.67)	**0.85 (0.83–0.86)**	0.80 (0.79–0.82)	0.77 (0.75–0.78)	0.69 (0.67–0.70)
(A1’) 80% training, 20% test, randomly selected ***patients*, *with RFE***						
XGBoost	**0.81 (0.80–0.82)**	**0.70 (0.69–0.72)**	0.83 (0.82–0.84)	**0.81 (0.80–0.82)**	**0.79 (0.77–0.80)**	**0.69 (0.67–0.70)**
Logistic Regression	0.77 (0.76–0.78)	0.62 (0.61–0.64)	**0.87 (0.86–0.88)**	0.80 (0.79–0.81)	0.74 (0.72–0.75)	0.69 (0.67–0.70)
(A2) 80% training, 20% test, randomly selected ***hospitals***						
XGBoost	**0.84 (0.83–0.85)**	**0.69 (0.67–0.70)**	0.85 (0.84–0.86)	**0.86 (0.85–0.87)**	**0.87 (0.86–0.88)**	0.64 (0.62–0.65)
Logistic Regression	0.81 (0.80–0.82)	0.62 (0.61–0.63)	**0.89 (0.88–0.90)**	0.86 (0.85–0.87)	0.83 (0.82–0.84)	**0.67 (0.66–0.69)**

### MIMIC-IV (B)

We carried out two experiments using the MIMIC-IV dataset: (B1) and (B1’) are similar to those in (A1) and (A1’). The RFE resulted in 132 relevant features (49 clinical variables).

Performance metrics are presented in Table 3. Fig 4C/4D illustrates XGBoost, which is demonstrated to outperform the LR method (baseline) in most of the metrics. Considering the AUROC, both experiments perform comparably, showing that using a reduced set of features, AUROC does not improve. However, experiment (B1’) shows lower numbers in Recall, F1-score, and NPV, indicating a worsened prediction using the RFE algorithm. The variations in the metrics are also reflected in the calibration curve in Fig 4D. When comparing the model’s performance between MIMIC-IV and eICU-CRD, it is observed that the former exhibits a comparatively lower AUROC by 0.18. In the subgroup analysis, the models for age, sepsis, and Horowitz index ≥162 had worse results compared to our main analysis.

**Table 3:** Model performance using the MIMIC-IV dataset. Comparison of selected metrics for XGBoost and LR (baseline) on the defined experiments, see Fig 3. The best-performing metric values are represented in bold.

Experiment	AUROC (95% CI)	AUPRC (95% CI)	Recall (95% CI)	F1-score (95% CI)	PPV (95% CI)	NPV (95% CI)
(B1) 80% training, 20% test, randomly selected ***patients***						
XGBoost	**0.65 (0.63–0.67)**	**0.81 (0.79–0.82)**	0.91 (0.89–0.92)	**0.52 (0.49–0.54)**	**0.36 (0.34–0.38)**	0.86 (0.85–0.88)
Logistic Regression	0.65 (0.63–0.67)	0.80 (0.79–0.82)	**0.99 (0.99–1.00)**	0.49 (0.46–0.51)	0.30 (0.30–0.34)	**0.93 (0.92–0.94)**
(B1’) 80% training, 20% test, randomly selected ***patients*, *with RFE***						
XGBoost	**0.66 (0.64–0.68)**	**0.81 (0.79–0.83)**	0.42 (0.40–0.44)	0.44 (0.41–0.46)	**0.45 (0.43–0.47)**	0.74 (0.72–0.76)
Logistic Regression	0.64 (0.62–0.66)	0.80 (0.78–0.81)	**0.52 (0.49–0.54)**	**0.47 (0.45–0.49)**	0.43 (0.41–0.45)	**0.76 (0.74–0.77)**

### Combined eICU-CRD and MIMIC-IV (C)

To better evaluate the robustness of our models against variance of data, we carried out six experiments using a combination of both datasets. We trained all models on a combination of both datasets but validated on a combination of both datasets (C1), on eICU-CRD (C2), and on MIMIC-IV (C3). For each of these experiments, we carried out experiments (C1’- C3’) using the RFE algorithm. The 18 common features (14 clinical variables) of both single dataset experiments (A1’) and (B1’) were considered to be used for the analysis of the combined dataset.

The corresponding performance metrics are presented in Table 4. As depicted in Table 4, XGBoost showed statistically significant superiority over the LR method with a 95% CI (baseline) in most experiments. The AUROC using XGBoost and associated calibration curves is illustrated in Fig 4E/4F. Compared to the test on MIMIC-IV (C3), the AUROC is similar while validating on the combined data set (C1) and on eICU-CRD (C2), reflected by the curve in Fig 4E. Considering the reduced set of features using the RFE algorithm (C1’) and (C2’), the AUROC is slightly inferior compared to the full set of features (C1) and (C2). Evaluating on eICU-CRD (C2) and (C2’) outperformed the one on MIMIC-IV (C3) and (C3’). Training and test solely on eICU-CRD (A1) and (A1’) slightly outperform training on the combined data and test on eICU-CRD (C2) and (C2’). In contrast, training on the combined data set leads to slightly better test in MIMIC-IV (C3) compared to training solely on MIMIC-IV (B1). Fig 4F shows the calibration curve of XGBoost; in this context, all experiments are well calibrated except (C3’) including MIMIC validation. In the subgroup analysis, the models for age and sepsis had worse results, only the subgroup with a Horowitz index ≥162 had a slightly better AUC compared to the main analysis.

**Table 4:** Model performance using the combined dataset. Comparison of selected metrics for XGBoost and LR (baseline) on the defined experiments, see Fig 3. The best-performing metric values are represented in bold font.

Experiment	AUROC (95% CI)	AUPRC (95% CI)	Recall (95% CI)	F1-score (95% CI)	PPV (95% CI)	NPV (95% CI)
(C1) 80% training, 20% test, randomly selected patients, ***test on combined data***						
XGBoost	**0.81 (0.80–0.82)**	**0.78 (0.77–0.79)**	0.68 (0.67–0.70)	**0.73 (0.72–0.74)**	**0.78 (0.77–0.79)**	0.70 (0.69–0.71)
Logistic Regression	0.78 (0.77–0.79)	0.72 (0.71–0.73)	**0.72 (0.70–0.73)**	0.72 (0.71–0.74)	0.73 (0.72–0.75)	**0.70 (0.69–0.71)**
(C1’) 80% training, 20% test, randomly selected patients**, *test on combined data***, ***with RFE***						
XGBoost	**0.78 (0.77–0.79)**	**0.75 (0.74–0.76)**	0.65 (0.64–0.67)	**0.71 (0.70–0.72)**	**0.77 (0.76–0.78)**	0.68 (0.67–0.69)
Logistic Regression	0.74 (0.72–0.75)	0.67 (0.66–0.68)	**0.73 (0.72–0.74)**	0.70 (0.69–0.71)	0.68 (0.66–0.69)	**0.68 (0.67–0.70)**
(C2) 80% training, 20% test, randomly selected patients, ***test on eICU-CRD***						
XGBoost	**0.82 (0.81–0.83)**	**0.72 (0.71–0.74)**	0.92 (0.92–0.93)	**0.81 (0.80–0.83)**	**0.73 (0.71–0.74)**	**0.76 (0.75–0.78)**
Logistic Regression	0.79 (0.77–0.80)	0.65 (0.64–0.67)	**0.94 (0.93–0.95)**	0.79 (0.77–0.80)	0.68 (0.66–0.69)	0.72 (0.70–0.73)
(C2’) 80% training, 20% test, randomly selected patients**, *test on eICU-CRD***, ***with RFE***						
XGBoost	**0.79 (0.77–0.80)**	**0.67 (0.66–0.69)**	0.92 (0.91–0.93)	**0.80 (0.79–0.81)**	**0.71 (0.69–0.72)**	**0.72 (0.71–0.74)**
Logistic Regression	0.75 (0.74–0.76)	0.61 (0.60–0.63)	**0.95 (0.94–0.96)**	0.78 (0.77–0.79)	0.66 (0.65–0.68)	0.68 (0.67–0.70)
(C3) 80% training, 20% test, randomly selected patients, ***test on MIMIC-IV***						
XGBoost	**0.67 (0.65–0.69)**	**0.82 (0.81–0.84)**	**0.56 (0.54–0.58)**	**0.48 (0.46–0.50)**	**0.42 (0.40–0.45)**	**0.76 (0.75–0.78)**
Logistic Regression	0.63 (0.60–0.65)	0.77 (0.75–0.79)	0.52 (0.50–0.54)	0.46 (0.44–0.48)	0.41 (0.39–0.43)	0.75 (0.73–0.77)
(C3’) 80% training, 20% test, randomly selected patients**, *test on MIMIC-IV***, ***with RFE***						
XGBoost	**0.64 (0.62–0.66)**	**0.80 (0.78–0.81)**	0.59 (0.56–0.61)	**0.49 (0.46–0.51)**	**0.42 (0.39–0.44)**	**0.77 (0.75–0.78)**
Logistic Regression	0.53 (0.51–0.56)	0.71 (0.69–0.73)	**0.69 (0.67–0.71)**	0.44 (0.42–0.46)	0.33 (0.31–0.35)	0.71 (0.69–0.73)

### Variable ranking

**Fig 5:**
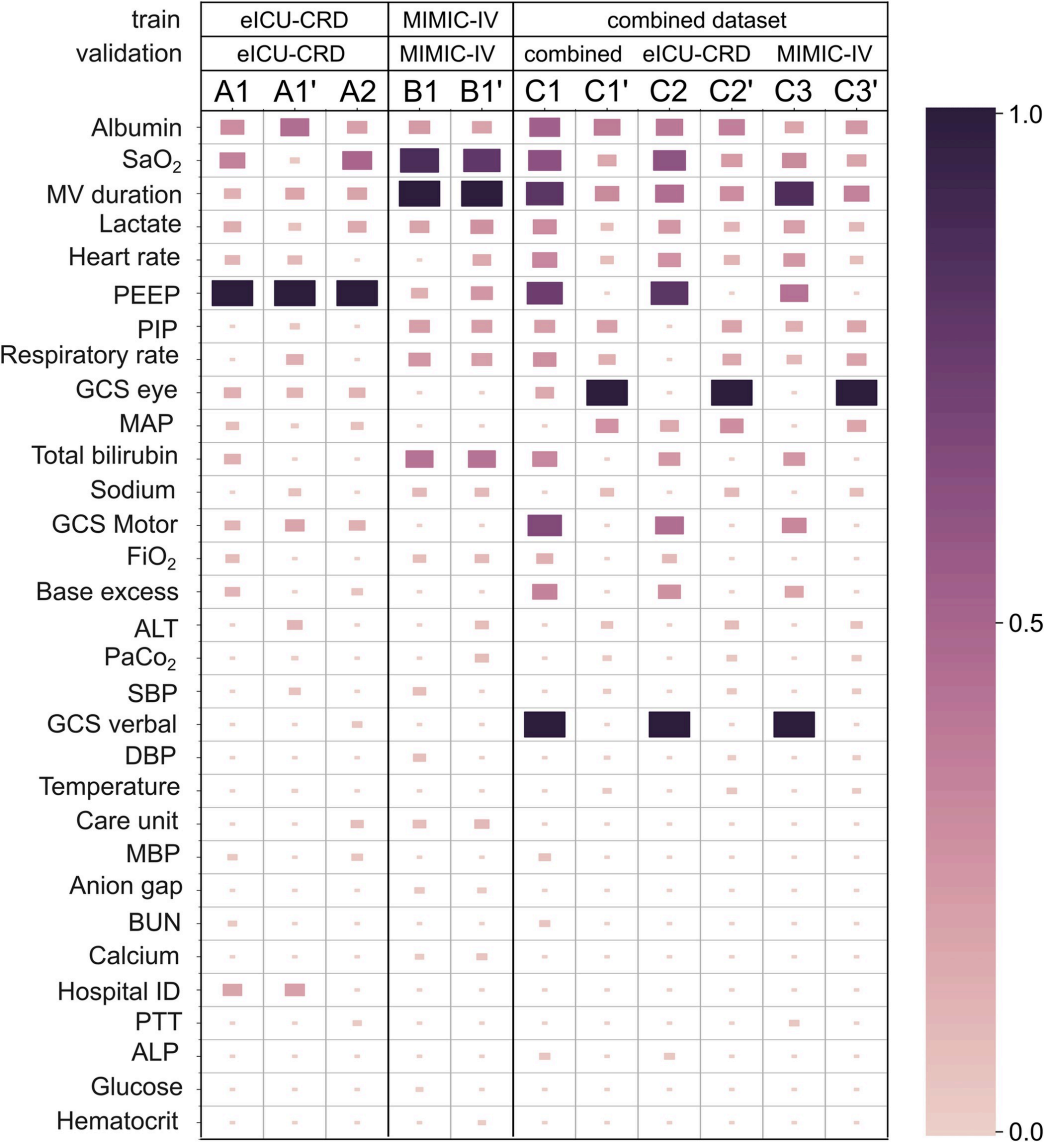
Variable ranking of the XGBoost experiments. A higher variable importance in XGBoost as based on the SHAP results is represented by a larger size and darker color of the rectangle. (ALP, alkaline phosphatase; ALT, alanine transaminase; BUN, blood urea nitrogen; DBP, diastolic blood pressure; FiO_2_, fraction of inspired oxygen; GCS, Glasgow Coma Scale; MAP, mean arterial pressure; MBP, mean blood pressure; MV, mechanical ventilation; PaCO_2_, partial pressure of carbon dioxide; PEEP, positive end-expiratory pressure; PIP, peak inspiratory pressure; PTT, partial thromboplastin time; SaO_2_, oxygen saturation; SBP, systolic blood pressure).

Application of ML models in medicine requires a reasonable justification for any recommendations [32]. Thus, we provided a visualized explanation of the XGBoost model on the level of the clinical variable. Fig 5 illustrates the most important variables with color and block size coding of importance and is based on the SHAP results. Associated individual beeswarm figures are presented in the S1 Appendix. While the variable importance differs between experiments (see S1 Appendix), serum albumin, serum lactate, SaO_2_, and MV duration are common among top-20 ranked variables for all experiments. Heart rate, GCS score (eye, motor, and verbal components) were among the top-20 variables for 9 out of 11 experiments. PEEP, and PIP are among the top-20 variables for 8 out of 11 experiments. SHAP values ranking depicts that the most ranked variable set highly depends on the employed data in the experiment.

## Discussion

### Principal findings

This study describes and validates a method to predict successful weaning from MV in critically ill patients. The prediction model was based on the PEEP-level, which has not been used in this form before. The trained model on the whole set of clinical variables performs well on the eICU-CRD dataset, but worse on the MIMIC-IV dataset, which can be improved to a degree by training with a joint dataset of MIMIC-IV and eICU-CRD. The predictive performance metrics are consistent, with only slight fluctuations in the experiments on one dataset. AUROC and AUPRC metrics show much higher fluctuations in the combined dataset experiments (C1—C3 and C1’—C3’) with respect to single dataset experiments. We divided the eICU-CRD dataset by hospitals, which constitutes a form of hospital-external validation within the dataset. We further provide insights into the approach and the key variables illustrated using an Interpretable ML method.

### Experimental observations

XGBoost consistently exhibits stable metrics for AUROC, AUPRC, and F1-score across the experiments of each single dataset, with this trend being prominently noticeable in Table 2 and also in Table 3. The stability in key performance metrics, with the AUROC combining specificity and sensitivity, the AUPRC emphasizing the model’s performance when dealing with imbalanced datasets, and the F1-score harmonizing precision and recall (being perfect with a value of 1.0), underscores the robustness of XGBoost in different experimental contexts. However, the PPV and NPV values depict a different scenario, showing considerable variability across different experimental settings without presenting a clear trend of increase or decrease. The PPV describes the ratio of predicting positives in the population with truly positive outcomes and is a little higher in the experiments on the eICU-CRD data than the NPV describing the ratio of predicting negatives in the population with truly negative outcomes. In the MIMIC data, the PPV is generally lower than the NPV, having a larger difference compared to eICU-CRD. This variability underscores the sensitivity of these metrics, which predict the positive test in the population with truly positive results and vice versa, to the specifics of the experimental design and underscores the need for careful consideration in their evaluation and interpretation.

Particular attention is required for the results using the combined dataset in Table 4, where more meaningful variations are observed across all metrics similar to experiments on individual datasets, especially in experiments C3 and C3’. These fluctuations signify the potential impact of different data contexts and experimental conditions on the performance metrics of the XGBoost model.

### Feature ranking

The features that SHAP identified to be influential on the outcome are consistent with known risk factors for weaning or extubation failure. A systematic review and meta-analysis by Li et al. [33] identified low serum albumin, low SaO_2_, shock (for which high lactate can act as a surrogate parameter), and length of mechanical ventilation as risk factors for reintubation. Those factors were among the top 20-ranked variables for all our experiments.

In addition to low serum albumin, Wu et al. [34] identified lower GCS scores as predictors for weaning failure in a retrospective observational study, a parameter that also ranked top 20 in 9 out of 11 experiments. Torrini et al. [35] were able to confirm the duration of mechanical ventilation, heart rate, and lower GCS scores as risk factors for unsuccessful extubation and weaning failure, whereas Na et al. [36] discovered higher peak inspiratory pressures (PIP) in patients with prolonged weaning. Our experiments also identified PIP as a possible risk factor for weaning failure.

Overall, all features identified by SHAP are congruent with those already associated with weaning failure by several studies thus increasing the plausibility of our results.

### Related work and design comparison

In this study, our algorithm is defined as a binary classification machine learning problem. It detects weaning from MV using changes in the PEEP level. In doing so, we predict weaning as a multistage process. The model design and patient cohort of our study are different from the previous studies. In this respect, a complete comparison is not viable, although the overall goal of predicting weaning can be contrasted to some extent.

Most related work relies on the data from a single local hospital to define its patient collective [11,12,14,18,19]. The resulting models may be used as a basis to provide local ICU weaning dashboards. Some authors use publicly available datasets, i.e., mostly a combination of eICU-CRD and MIMIC-IV [16], a combination of a local hospital and a public dataset [20] or only one of those [15].

The number of features included into the modeling is widely varying in the related work, where the selection of features used in the final models is not always clear. Considering our best-performing model that used RFE algorithm, we included 20 clinical variables.

The prediction of a direct full weaning appears to achieve comparatively best performance. The full weaning algorithm described by Liao et. al. [14] incorporates 26 features (+6 to our best model), and results in a slightly better AUROC of 0.86. Similarly, Liu et. al. [16] used 35 features (+15 features) from the MIMIC-IV and eICU-CRD data to predict a full weaning. and achieved a better AUROC of 0.80 in the MIMIC-IV validation and 0.86 in the eICU-CRD validation. Jia et. al. [15] used 25 features (+5 features) from the MIMIC-III data to predict a full weaning achieving a high AUROC of 0.94. Otaguro et. al. [11] reported an AUROC of 0.95 with 57 features (+37 features). Lin et. al. [12] reported an AUROC of 0.90 using 300 features (+280 features).

Predicting an initial easing of weaning in a multi-stage process, or “try-weaning” as named by Liu et. al. [18], usually achieves lower scores than directly predicting the full weaning. There is only little work done on multi-stage weaning. Liu et. al. [18] used 25 features (+5 features) and achieved an slightly higher AUROC of 0.85 for the first stage and for the second stage they achieved an AUROC of 0.90. Similarly, Cheng et. al [19] achieve a lower AUROC of 0.76 with 32 features (+12 features). Both studies reported comparable or slightly inferior results compared to our work.

### Influence of data differences

In our study, we did not restrict the input data to a fixed time frame before an event. Thus, each event is predicted using data from a varying observation window. Since the outcome is defined solely based on the PEEP parameter and aggregated statistical measures, the number of events depends on the frequency of measurements in each dataset. Thus, it is of importance that PEEP measurements in our eICU-CRD cohort are documented on average (SD) every 38 (155) minutes and on median every 19 minutes. In contrast, in MIMIC-IV measurements are reported on average (SD) every 57 (323) minutes and on median every 36 minutes. Another difference in our cohort data is the positive outcome rate, which differs greatly at 35% versus 70%.

Such differences in the data might explain the performance differences among the various datasets included in this study. Moreover, clinical variables of the two datasets have various frequencies depending on the method each variable was measured and recorded and the severity of its patient catchment area. This might result in different predictive and descriptive performance outcomes, especially with the usage of the statistical derivations of our model approach.

### Limitations

The proposed models are trained and evaluated using datasets from the US and, thus, are adapted to the US situation. We did not limit this study to patients with a specific disease and therefore can predict weaning in a wide range of diseases. A more specific cohort of patients might result in models with better performance metrics.

## Conclusion

Weaning from MV, defined by a decrease in the positive end-expiratory pressure, can be predicted using a few features from an observation window of varying length before weaning. As such, this study describes a new strategy for predicting weaning in a multi-stage critical care decision-making process. In particular, the context of modeling and diversity of measurement methodologies in the different hospitals might need to be investigated in greater depth.

## Supporting information

S1 Appendix Additional details on methods and results, including a sub-group analysis. (DOCX)
