# Supplementary material for: Predicting Successful Weaning from Mechanical Ventilation by Reduction in Positive End-expiratory Pressure Level Using Machine Learning
Source: PLOS Digit Health. 2024 Mar 27;3(3):e0000478. doi: 10.1371/journal.pdig.0000478 (PMC10971612; doi:10.1371/journal.pdig.0000478)
Supplement: S1 Appendix — (DOCX) [file pdig.0000478.s001.docx]

## S1 Appendix: Online Supplementary Data – Predicting Successful Weaning from Mechanical Ventilation by Reduction in Positive End-expiratory Pressure Level Using Machine Learning

### Methods

#### Data Preprocessing and variable selection

In MIMIC-IV, most of the clinical variables are extracted from "chart events" and "lab" tables. In eICU-CRD, most of the clinical variables are extracted from "lab", "respiratory charting", and "nurse charting" tables. We converted the extracted clinical variables to wide format and ordered them by the time they were recorded in the database.

**Table 1. The set of variables included in the study.**

| **Variable group** | **Variable name** |
| --- | --- |
| **Demographic data** | Age, Gender |
| **Vital signs** | FiO_2_, GCS Eye, GCS Motor, GCS Verbal, INR, MAP, PEEP, PTT, PT, PIP, Anion gap, Base excess, Diastolic BP, Mean BP, Systolic BP, Glucose, Heart rate, PaCo_2_, PaO_2_, Respiratory rate, SaO_2_, Temperature |
| **Laboratory Measurements** | ALP, ALT, AST, MCHC, MCH, MCV, RBC, RDW, WBC, Albumin, Bicarbonate, BUN, Calcium, Chloride, Creatinine, Hematocrit, Hemoglobin, Lactate, pH, Platelets, Potassium, Sodium, Total bilirubin |
| **Other Measurements** | Mechanical Ventilation Duration, Weight, Height |

#### Model Training and Evaluation

**Table 2. True/False-Positive/Negative metrics of all experiments.**

| **Experiment** | **True Positive** | **False Positive** | **True negative** | **False Negative** |
| --- | --- | --- | --- | --- |
| **A1** | **934 (24%)** | **513 (13%)** | **2,019 (52%)** | **416 (11%)** |
| **A1’** | **894 (23%)** | **553 (14%)** | **2,028 (52%)** | **407 (10%)** |
| **A2** | **882 (19%)** | **428 (9%)** | **2,798 (61%)** | **499 (11%)** |
| **B1** | **736 (35%)** | **715 (34%)** | **472 (22%)** | **191 (9%)** |
| **B1’** | **1,164 (55%)** | **287 (14%)** | **220 (10%)** | **443 (21%)** |
| **C1** | **2,297 (38%)** | **601 (10%)** | **2,154 (36%)** | **994 (16%)** |
| **C1’** | **2,225 (37%)** | **673 (11%)** | **2,072 (35%)** | **1,026 (17%)** |
| **C2** | **695 (18%)** | **752 (19%)** | **2,191 (56%)** | **244 (6%)** |
| **C2’** | **585 (15%)** | **862 (22%)** | **2,198 (57%)** | **237 (6%)** |
| **C3** | **1,027 (49%)** | **424 (20%)** | **339 (16%)** | **324 (15%)** |
| **C3’** | **985 (47%)** | **466 (22%)** | **330 (16%)** | **333 (16%)** |

### Results

**Table 3. Variable included in each experiment, where** ✓ **represents the presence of each variable.**

| **Base Variable** | **A1, A2** | **B1, C1, C2, C3** | **A1’** | **B1’** | **C1’, C2’, C3’** |
| --- | --- | --- | --- | --- | --- |
| Age | ✓ | ✓ | ✓ | ✓ |  |
| Albumin | ✓ | ✓ | ✓ | ✓ | ✓ |
| ALP | ✓ | ✓ |  | ✓ |  |
| ALT | ✓ | ✓ | ✓ | ✓ | ✓ |
| Anion gap | ✓ | ✓ |  | ✓ |  |
| AST | ✓ | ✓ |  | ✓ |  |
| Base excess | ✓ | ✓ |  | ✓ |  |
| Bicarbonate | ✓ | ✓ |  | ✓ |  |
| BUN | ✓ | ✓ |  | ✓ |  |
| Calcium | ✓ | ✓ |  | ✓ |  |
| Care unit | ✓ | ✓ |  | ✓ |  |
| Chloride | ✓ | ✓ |  | ✓ |  |
| Creatinine | ✓ | ✓ |  |  |  |
| Diastolic BP | ✓ | ✓ | ✓ | ✓ | ✓ |
| FiO_2_ | ✓ | ✓ |  | ✓ |  |
| GCS Eye | ✓ | ✓ | ✓ | ✓ | ✓ |
| GCS Motor | ✓ | ✓ | ✓ | ✓ |  |
| GCS Verbal | ✓ | ✓ |  |  |  |
| Gender | ✓ | ✓ |  | ✓ |  |
| Glucose | ✓ | ✓ |  | ✓ |  |
| Heart rate | ✓ | ✓ | ✓ | ✓ | ✓ |
| Height | ✓ | ✓ |  | ✓ |  |
| Hematocrit | ✓ | ✓ |  | ✓ |  |
| Hemoglobin | ✓ | ✓ |  | ✓ |  |
| INR | ✓ | ✓ |  | ✓ |  |
| Lactate | ✓ | ✓ | ✓ | ✓ | ✓ |
| MAP | ✓ | ✓ | ✓ | ✓ |  |
| MCH | ✓ | ✓ |  | ✓ |  |
| MCHC | ✓ | ✓ |  | ✓ |  |
| MCV | ✓ | ✓ |  | ✓ |  |
| Mean BP | ✓ | ✓ |  | ✓ |  |
| MV Duration | ✓ | ✓ | ✓ | ✓ | ✓ |
| PaCo_2_ | ✓ | ✓ | ✓ | ✓ | ✓ |
| PaO_2_ | ✓ | ✓ |  | ✓ |  |
| PEEP | ✓ | ✓ | ✓ | ✓ |  |
| pH | ✓ | ✓ |  | ✓ |  |
| PIP | ✓ | ✓ | ✓ | ✓ | ✓ |
| Platelets | ✓ | ✓ |  | ✓ |  |
| Potassium | ✓ | ✓ |  | ✓ |  |
| PT | ✓ | ✓ |  | ✓ |  |
| PTT | ✓ | ✓ |  | ✓ |  |
| RBC | ✓ | ✓ |  | ✓ |  |
| RDW | ✓ | ✓ |  | ✓ |  |
| Respiratory rate | ✓ | ✓ | ✓ | ✓ | ✓ |
| SaO_2_ | ✓ | ✓ | ✓ | ✓ | ✓ |
| Sodium | ✓ | ✓ | ✓ | ✓ | ✓ |
| Systolic BP | ✓ | ✓ | ✓ | ✓ | ✓ |
| Temperature | ✓ | ✓ | ✓ | ✓ | ✓ |
| Total bilirubin | ✓ | ✓ | ✓ | ✓ |  |
| WBC | ✓ | ✓ |  | ✓ |  |
| Weight | ✓ | ✓ |  | ✓ |  |

#### Variable Ranking

Figure 1 to 3 shows detailed variable ranking using SHAP.

Considering eICU-CRD experiments (A1, A1’, A2), the most prominent clinical variables are PEEP, SaO_2_, serum albumin, GCS, eye and motor component subscores, fraction of inspired oxygen (FiO_2_), serum lactate, mean arterial pressure (MAP), and duration of MV. Similarly in MIMIC-IV experiments (B1, B1’) the most prominent clinical variables are serum albumin, type of assigned care unit, FiO_2_, total serum bilirubin, serum lactate, PEEP, PIP, SaO_2_, serum sodium, respiratory rate, and MV.

In the combined dataset before employing RFE algorithm (C1, C2, C3), the most important variables are serum albumin, base excess, GCS (motor, verbal), serum lactate, total serum bilirubin, heart rate, PEEP, SaO_2_, and MV. In the combined dataset after employing the RFE algorithm (C1’, C2’, C3’), the most important variables are serum albumin, the GCS eye component subscore, MAP, PIP, serum lactate, respiratory rate, heart rate, SaO_2_, and MV.


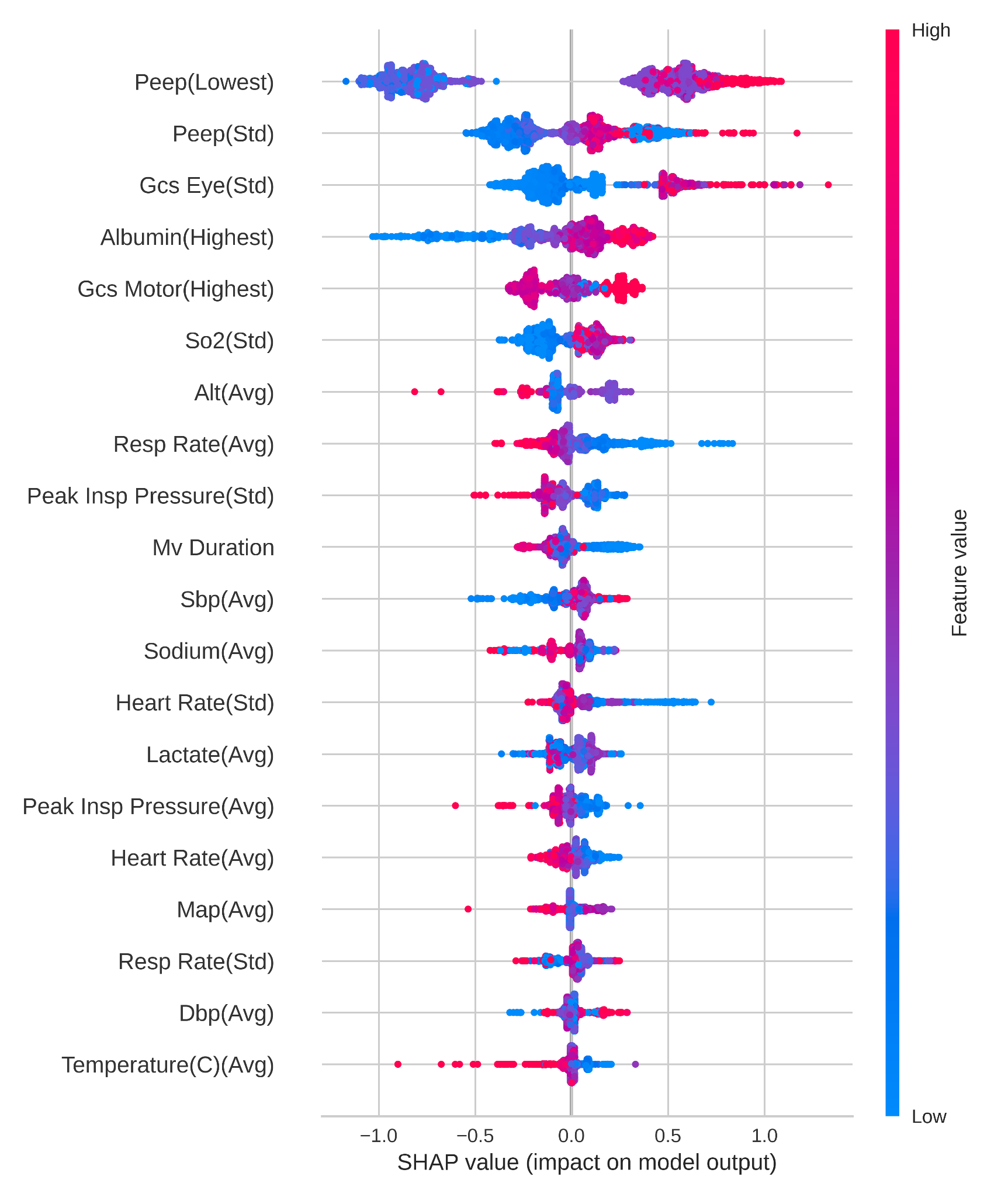

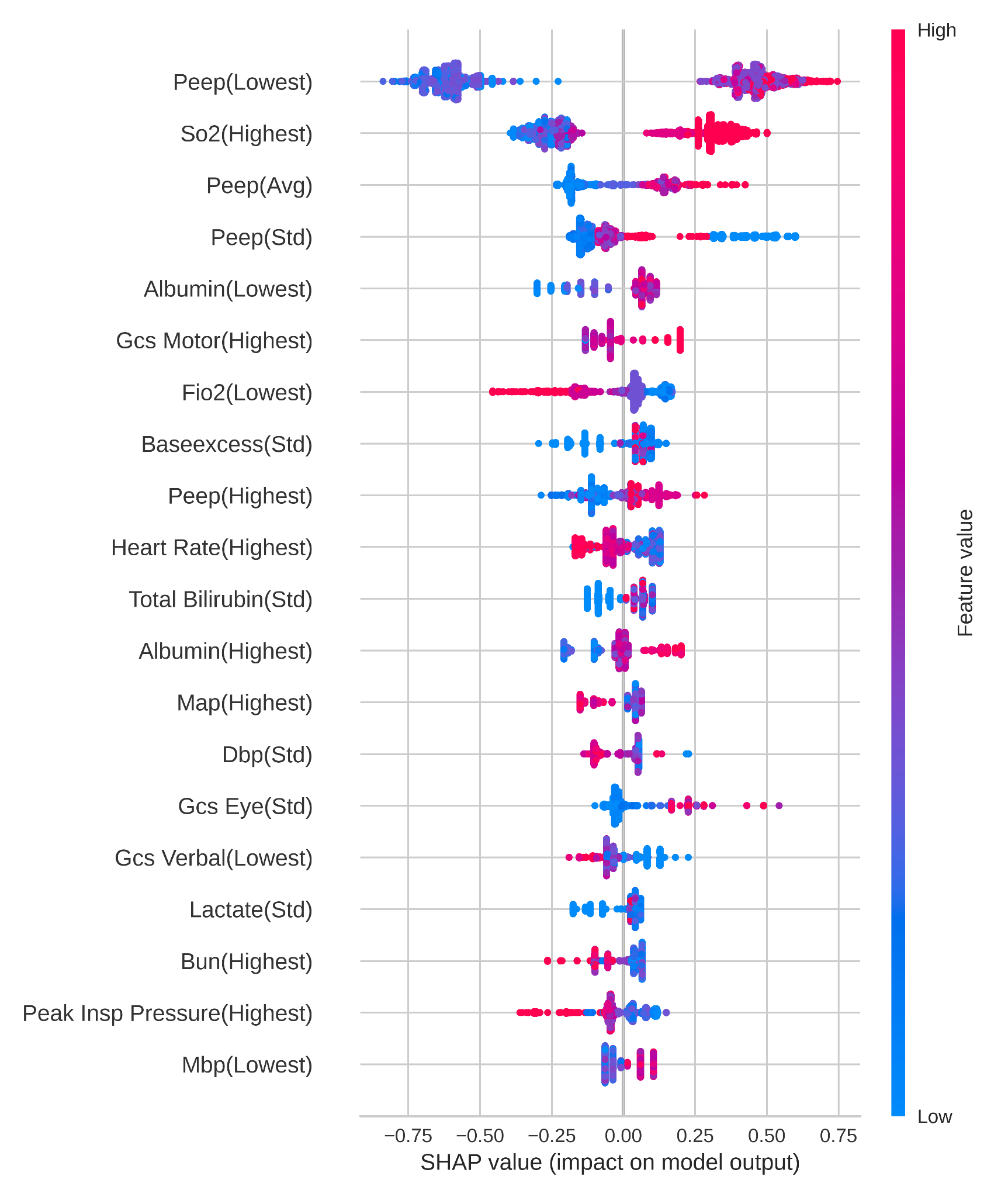


**A1 A1’**


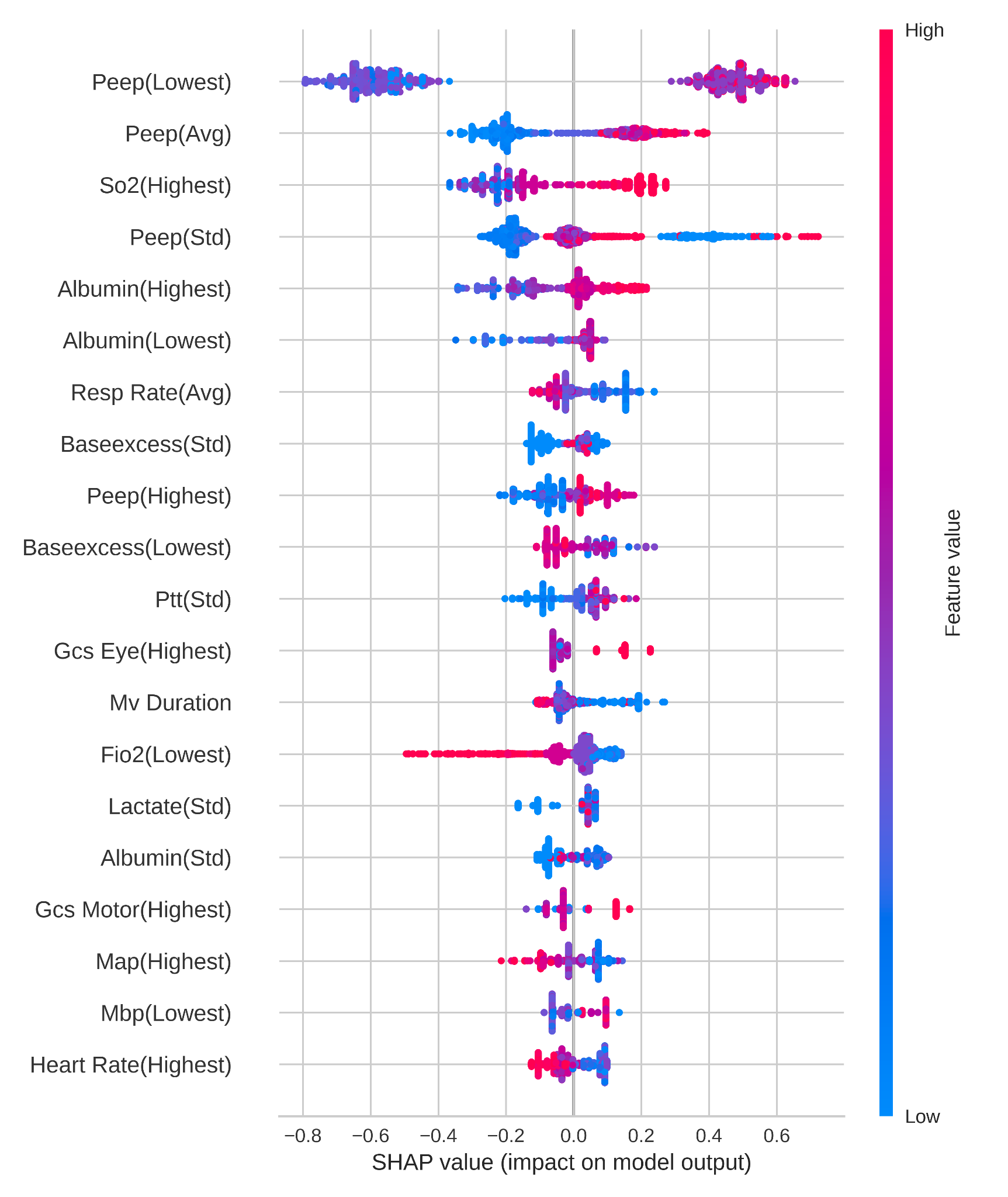


A2

**Figure 1. Variable ranking using SHAP for eICU-CRD experiments.** In each figure, the highest-ranked 20 variables are listed, and their importance diminishes from the top to the bottom.


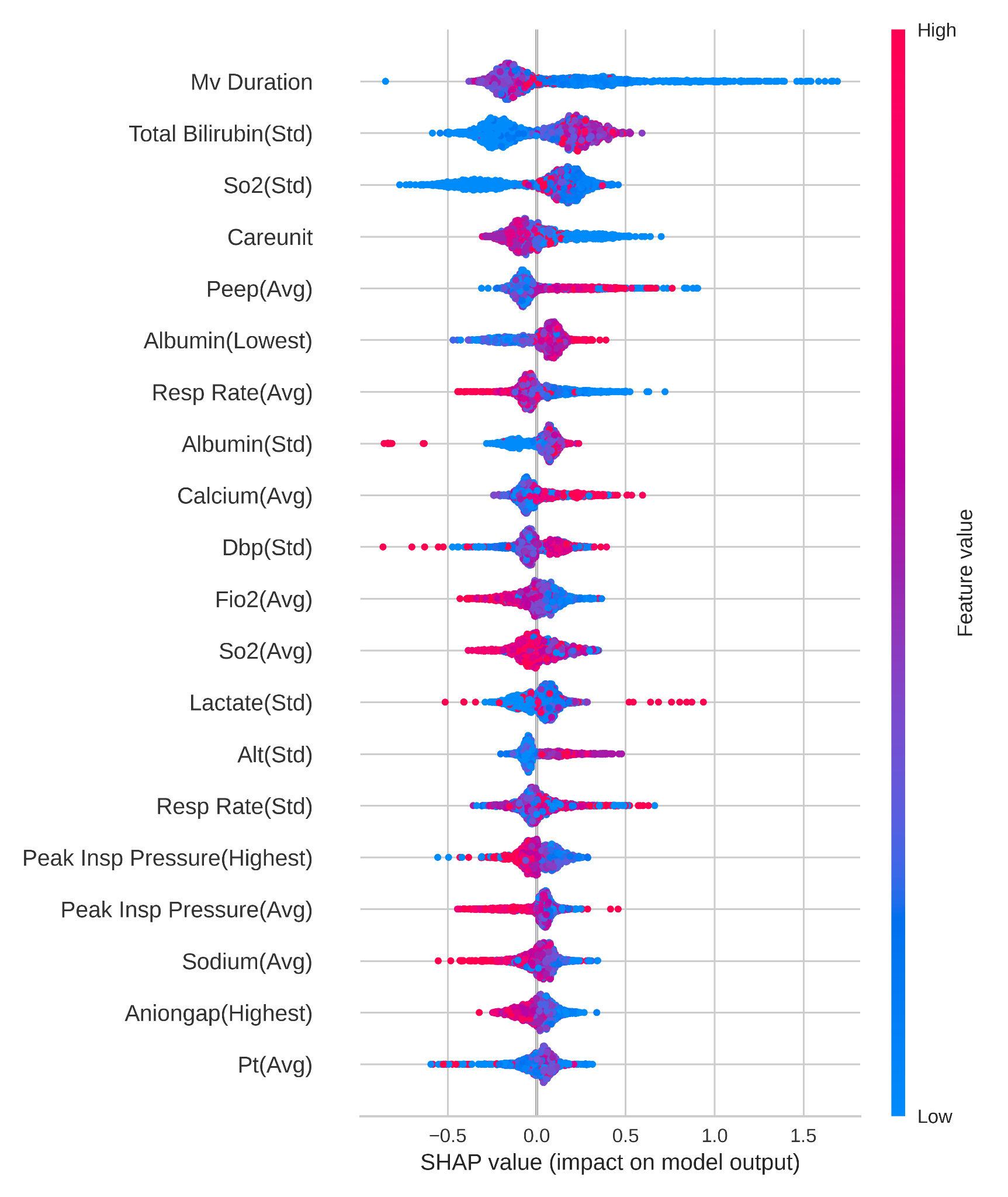

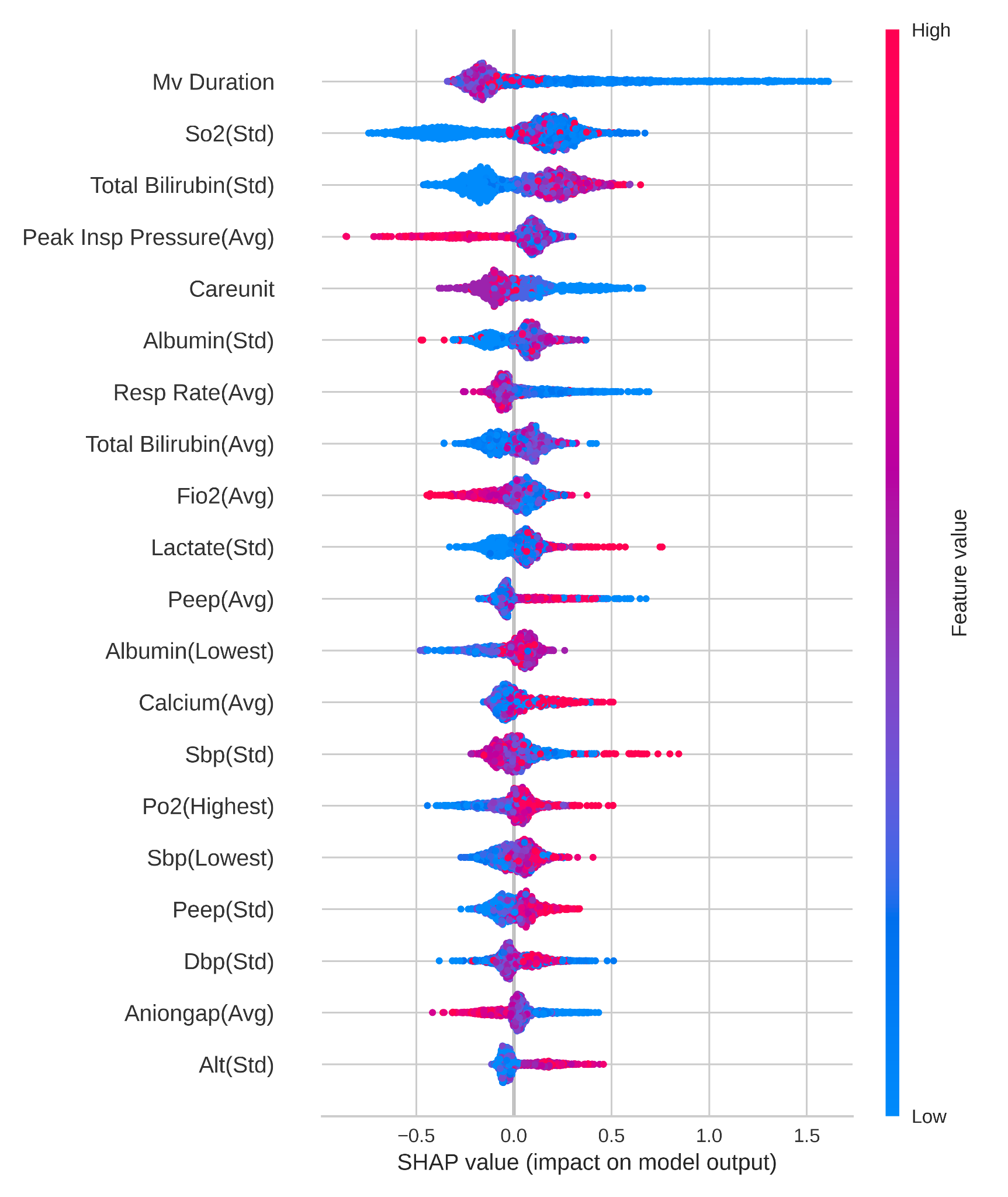


**B1 B1’**

**Figure 2. Variable ranking using SHAP for MIMIC-IV experiments.** In each figure, the highest-ranked 20 variables are listed, and their importance diminishes from the top to the bottom.


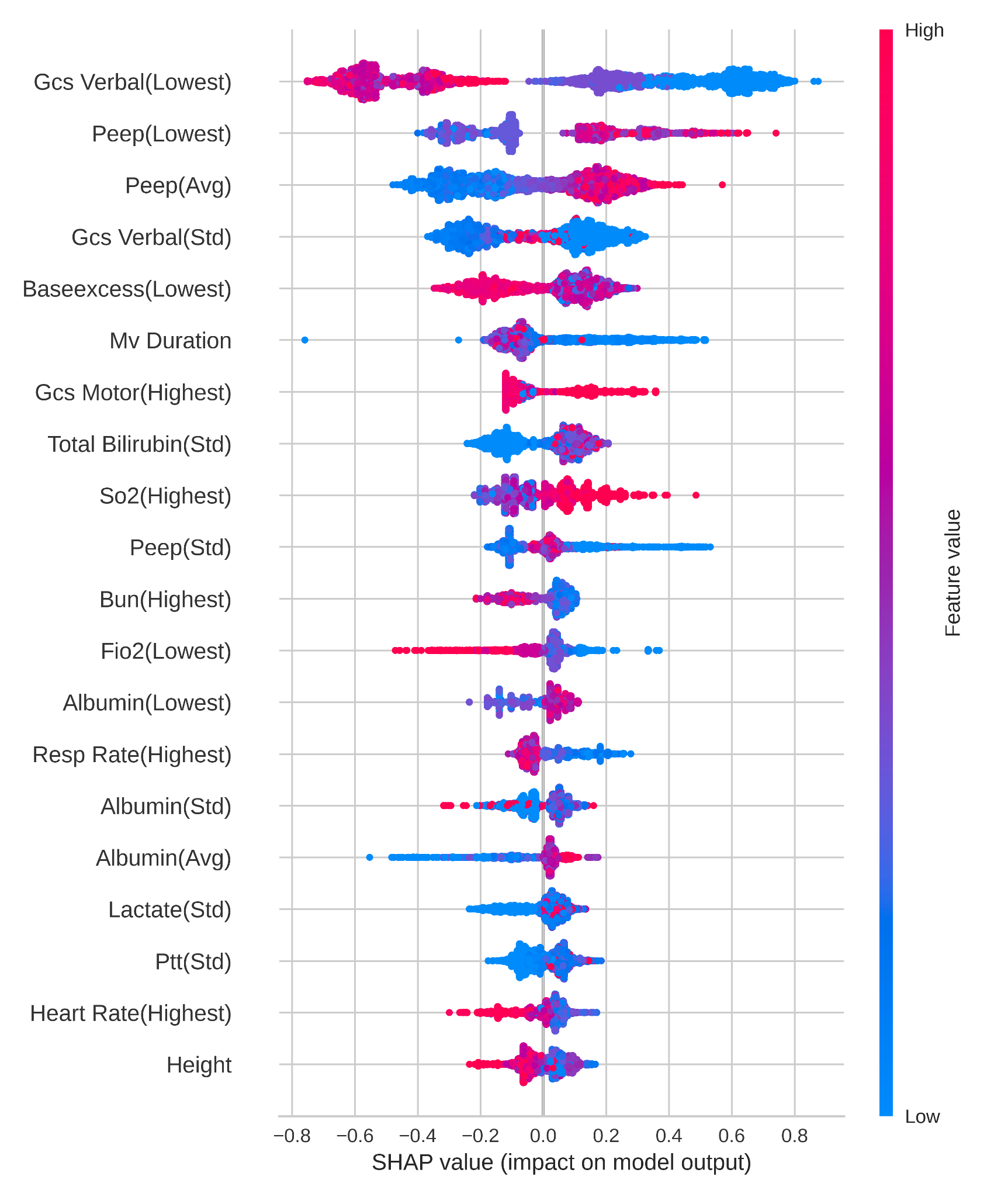

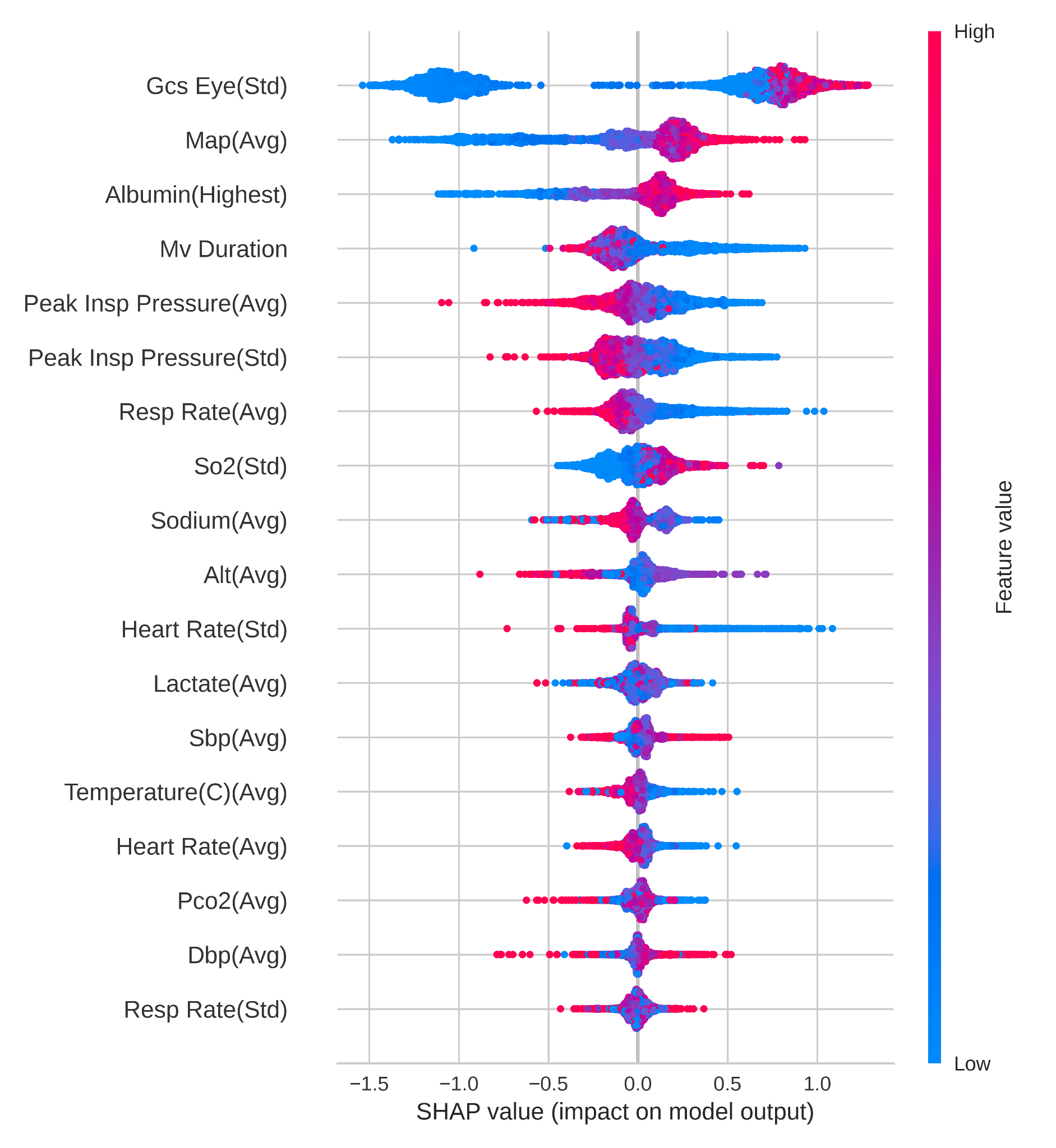
**C1 C1’**


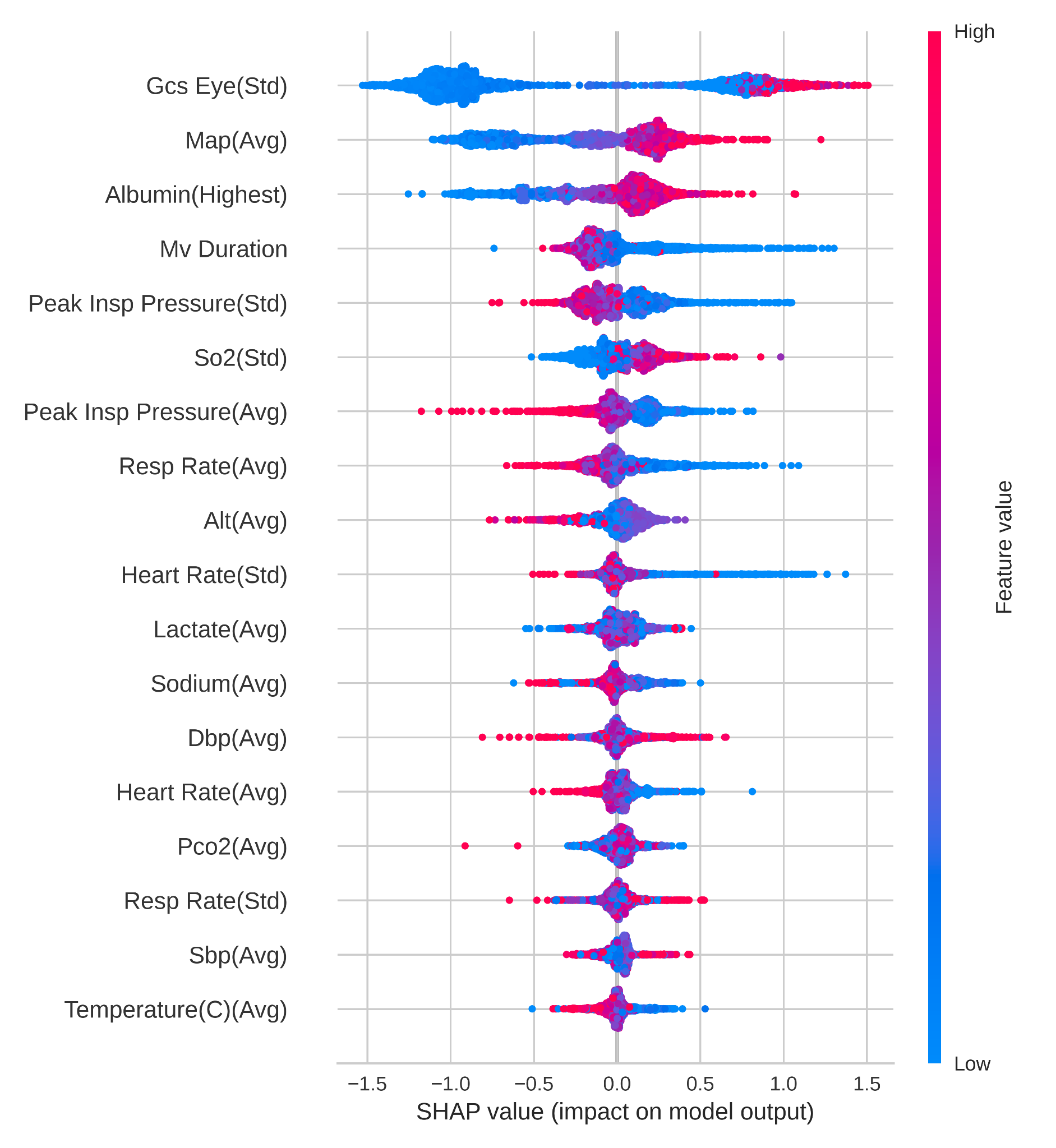

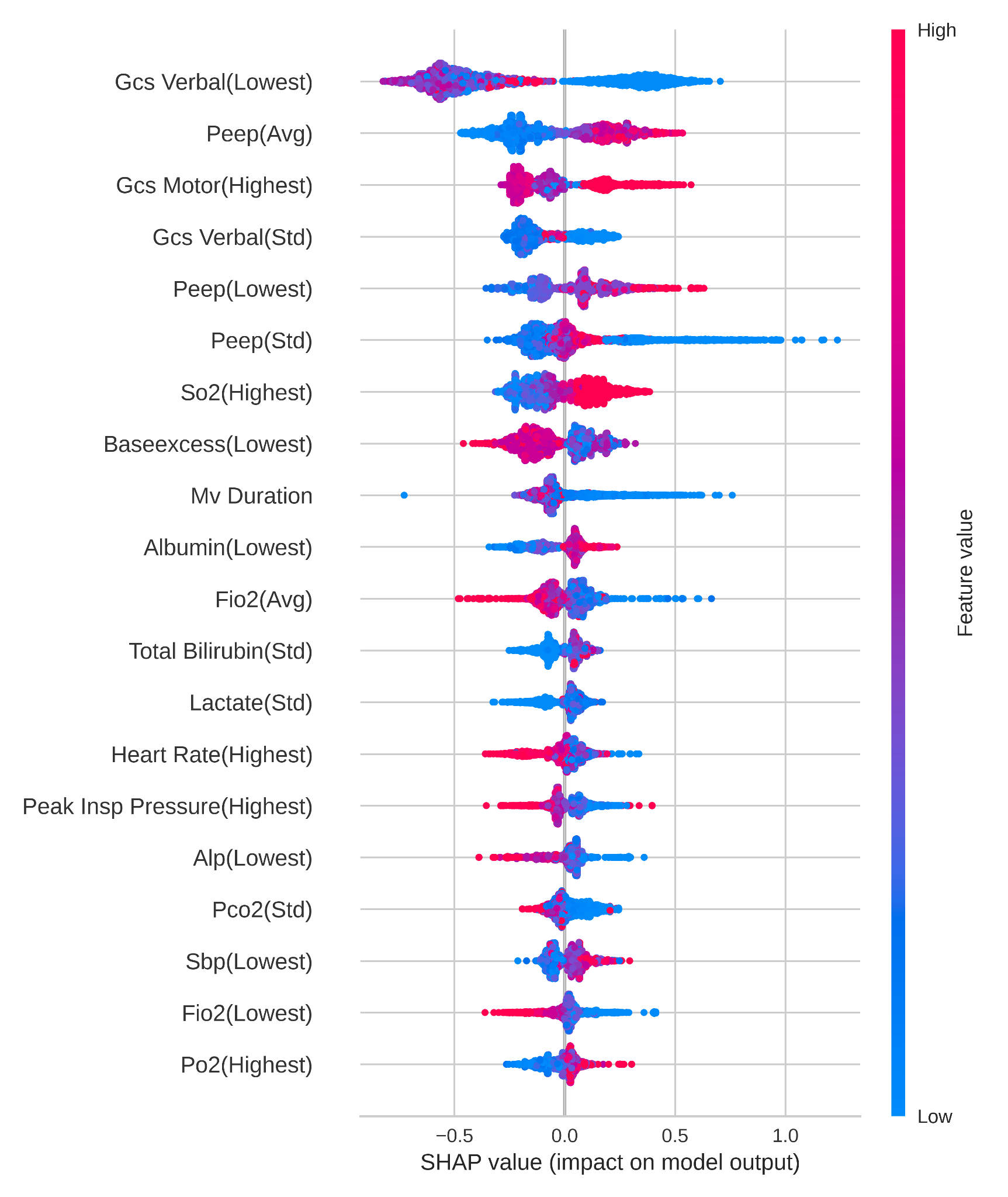


**C2 C2’**


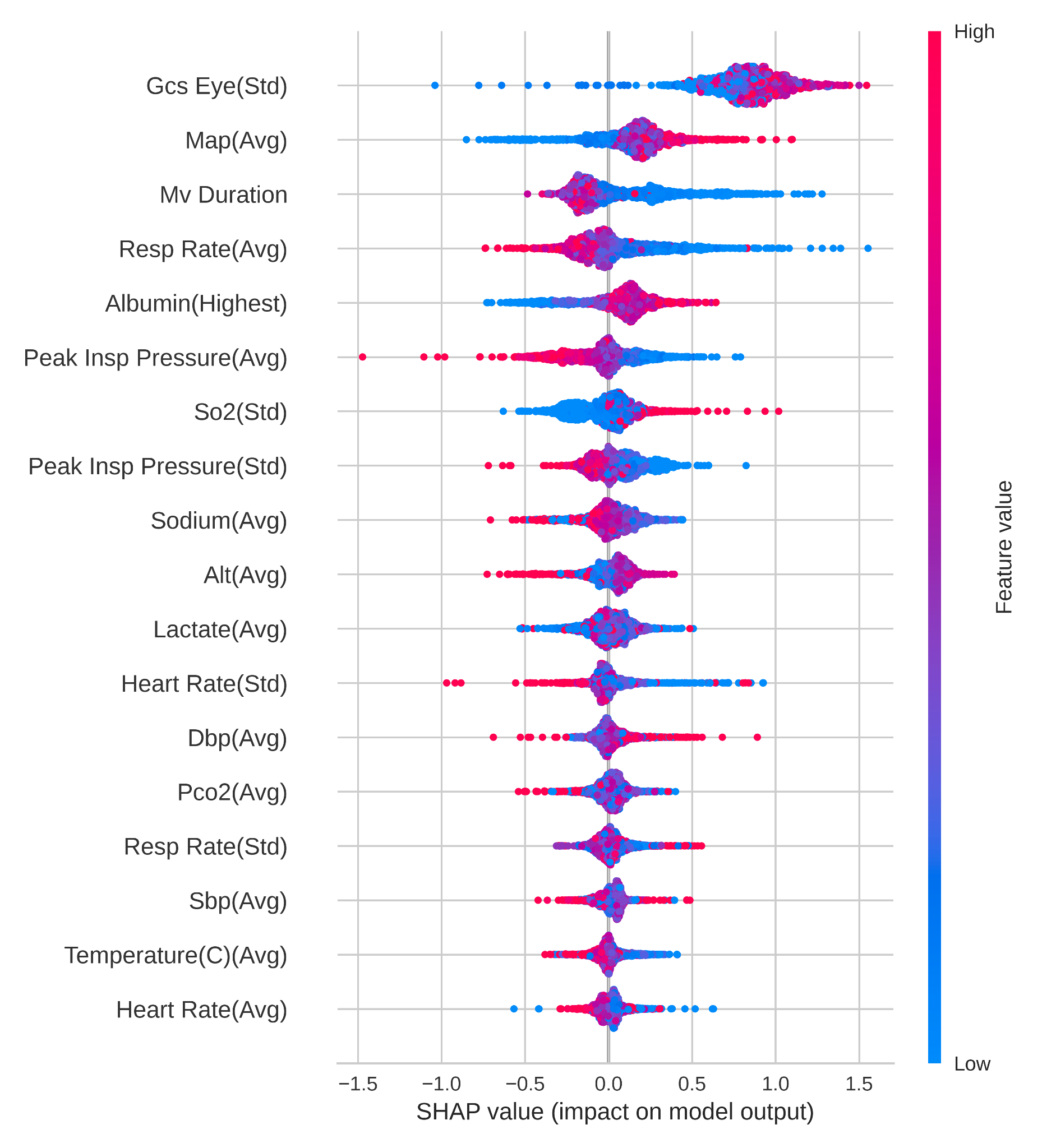


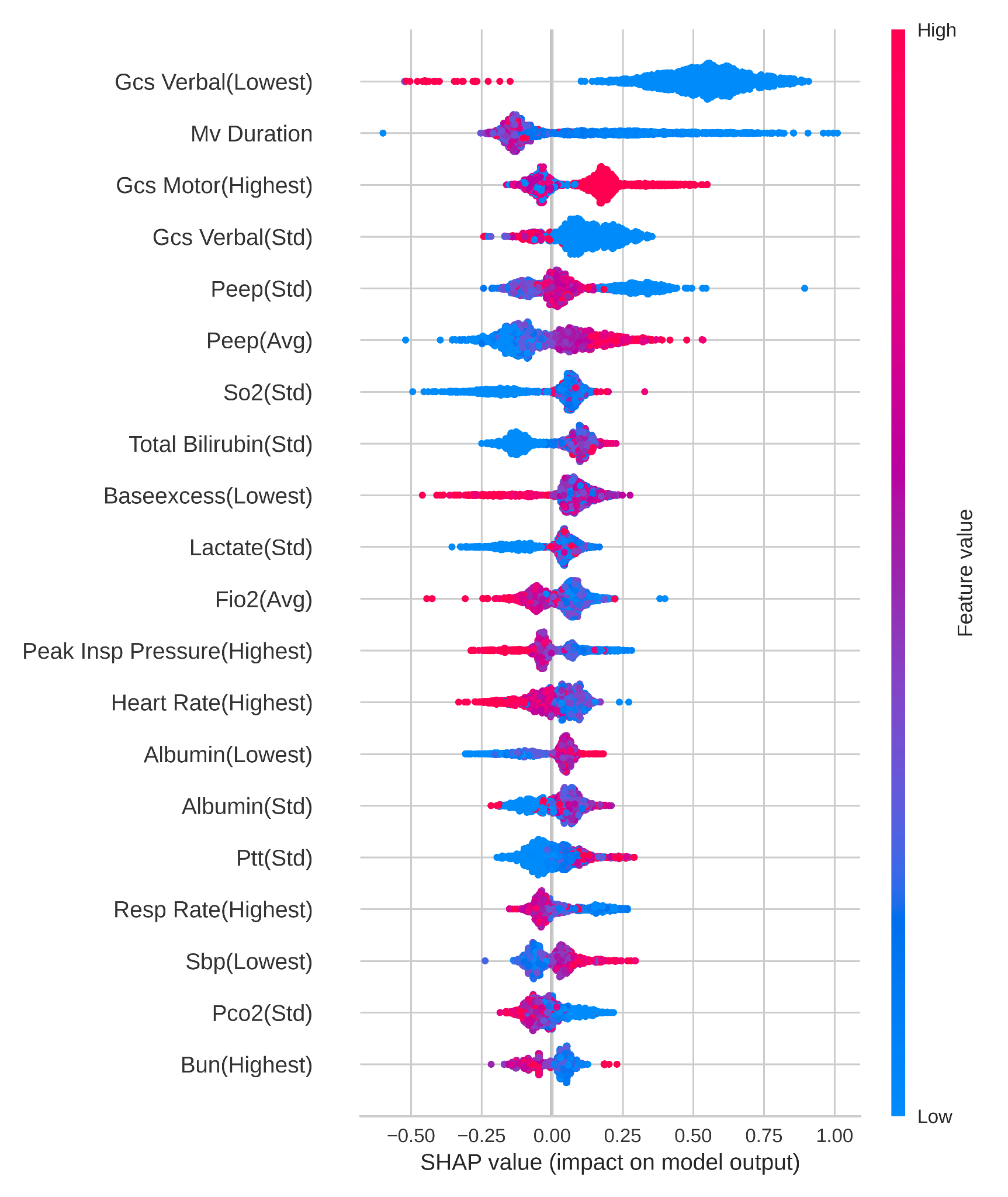


**C3 C3’**

**Figure 3. Variable ranking using SHAP for the combined experiments.** In each figure, the highest-ranked 20 variables are listed, and their importance diminishes from the top to the bottom.

### Sub-group analysis

The analysis described in the manuscript is intended to provide a prediction algorithm for the whole patient cohort using all available source data. To provide more insight into the cohort, we are providing a subgroup analysis. For this, the cohort was stratified by age, sepsis, and Horowitz index. Performance is described by AUROC and calibration curves.

#### Age

The overall cohort was stratified into three age groups: <50, 50-70, >70. The cohort aged <50 years, resulted in 1,153 patients for eICU-CRD and 1,196 patients for MIMIC-IV, the cohort aged 50-70 years resulted in 3,005 patients for eICU-CRD and 2,876 patients for MIMIC-IV, and the cohort aged >70 years resulted in 2,082 patients for eICU-CRD and 2,297 patients for MIMIC-IV.

As depicted in Figure 4, the model for patients aged 50-70 years outperformed the model for <50 years and >70 years. Accordingly, as shown in Figure 5, the model for patients aged 50-70 is better calibrated compared to two other subgroups.

#### Sepsis

The overall cohort was stratified by existence of sepsis. The selection of patients with sepsis resulted in 1,170 patients for eICU-CRD and 1,154 patients for MIMIC-IV. The selection for patients without sepsis (non-septic) resulted in 4,840 patients for eICU-CRD and 4,977 patients for MIMIC-IV. As depicted in Figure 6, the analysis showed a marked difference in predictive performance between the septic and non-septic subgroups. The model trained on non-septic patients exhibited a 1-10% higher accuracy in prediction weaning outcomes. This enhancement in performance for non-septic patients underscores the significant impact of sepsis on weaning outcomes.

#### Horowitz index

The overall cohort was stratified by median over the Horowitz index: <162 and ≥162. The median was calculated over the worst Horowitz indices of the first day of each encounter.

Patients with a Horowitz index <162 resulted in 2,771 patients for eICU-CRD and 3,242 patients for MIMIC-IV. Patients with an index <162 resulted in 2,520 patients for eICU-CRD and 2,889 patients for MIMIC-IV. For the first group, the model showed a 10% improvement in predictive performance as measured by AUROC and shown in Figure 8. The superior performance in the second group (higher Horowitz index) might be attributable to better baseline respiratory function, suggesting that patients with better initial respiratory status have more predictable weaning outcomes.

| **Age <50** | **Age 50-70** | **Age >70** |
| --- | --- | --- |
| 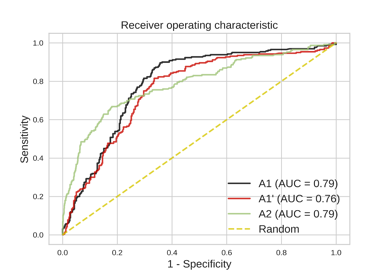 | 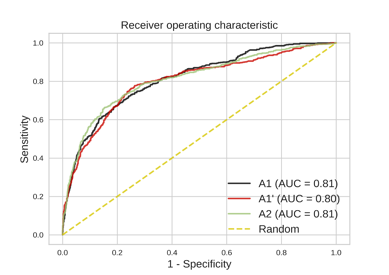 | 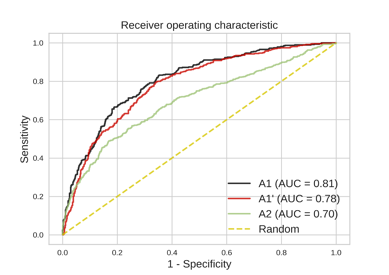 |
| 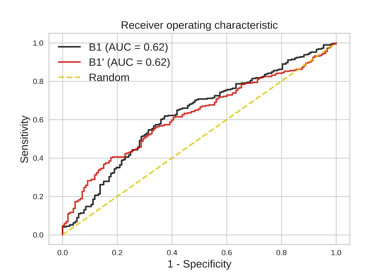 | 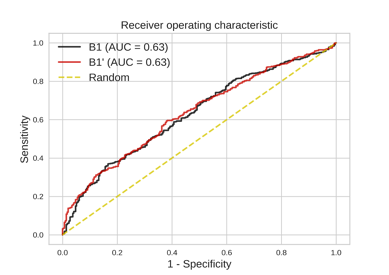 | 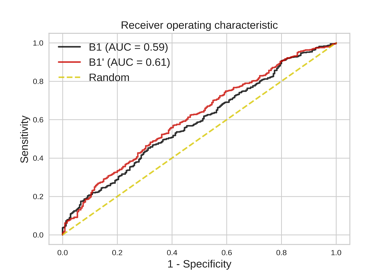 |
| 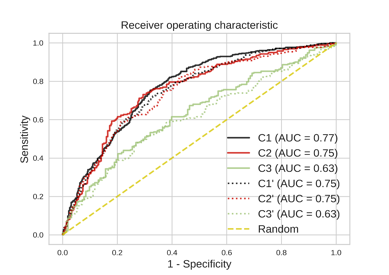 | 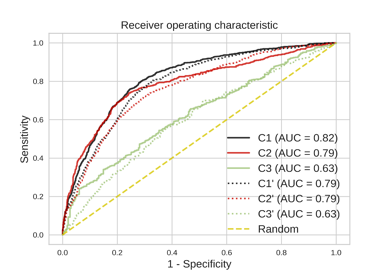 | 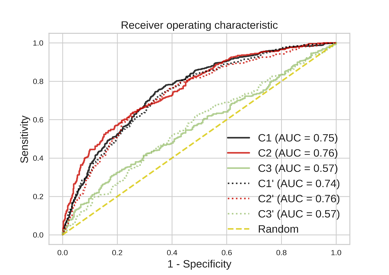 |

**Figure 4. AUROC of sub-group analysis based on age group using XGBoost.**

| **Age <50** | **Age 50-70** | **Age >70** |
| --- | --- | --- |
| 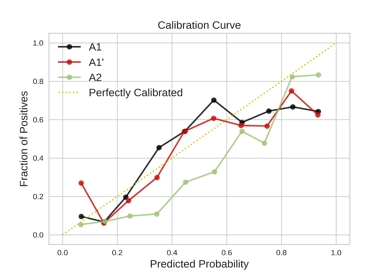 | 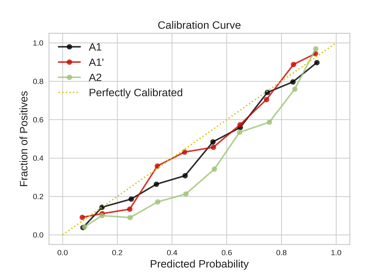 | 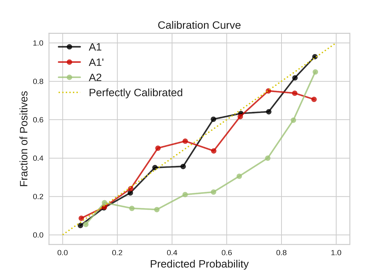 |
| 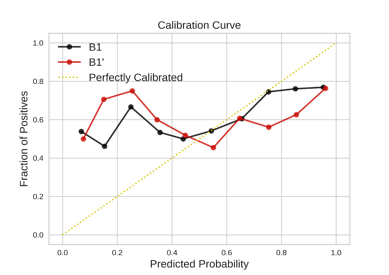 | 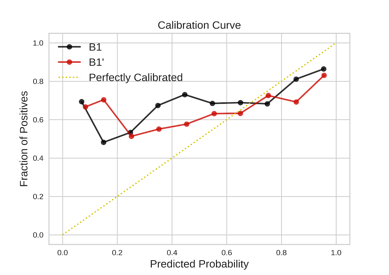 | 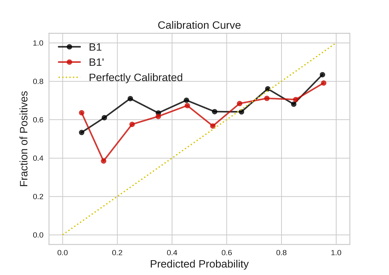 |
| 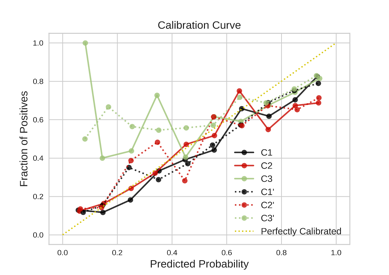 | 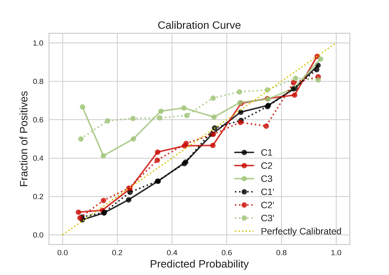 | 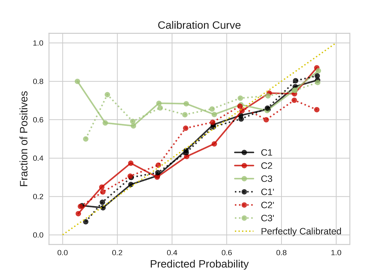 |

**Figure 5. Calibration curve of sub-group analysis based on age group using XGBoost.**

| **Septic** | **Non-septic** |
| --- | --- |
| 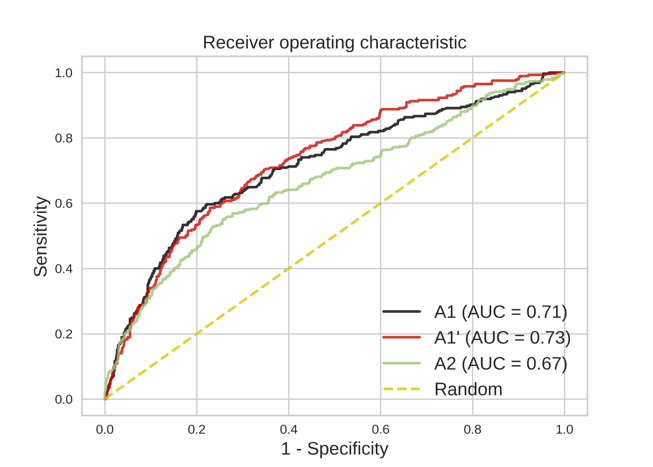 | 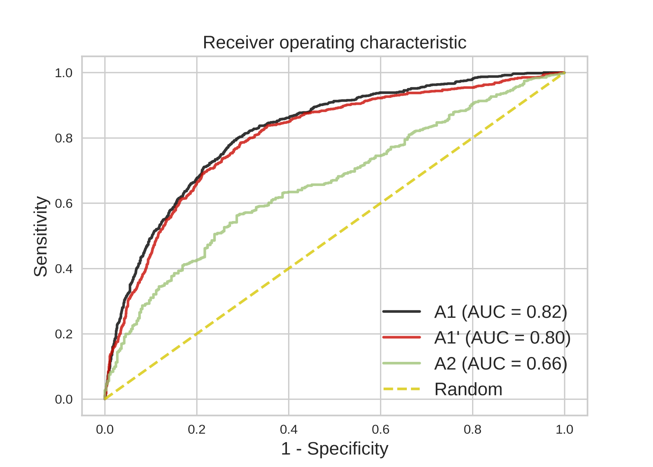 |
| 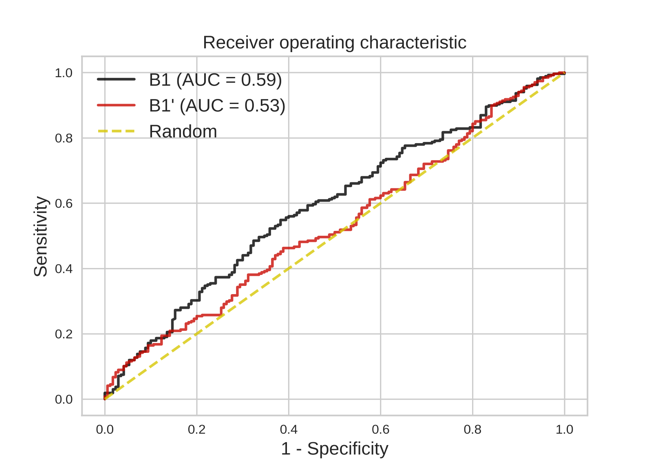 | 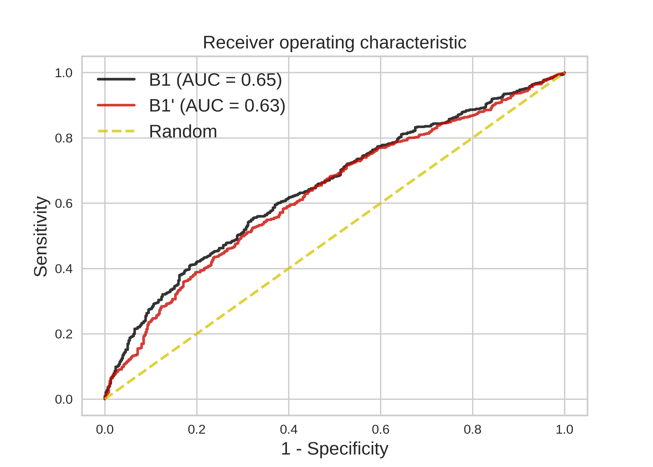 |
| 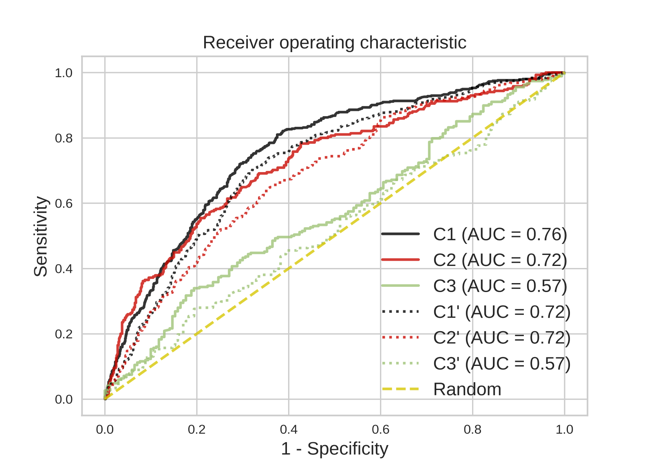 | 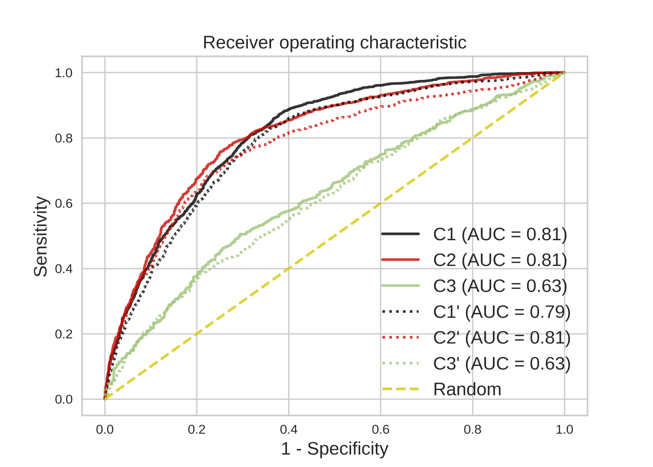 |

**Figure 6. AUROC of sub-group analysis of Septic vs. Non-septic patients using XGBoost.**

| **Septic** | **Non-septic** |
| --- | --- |
| 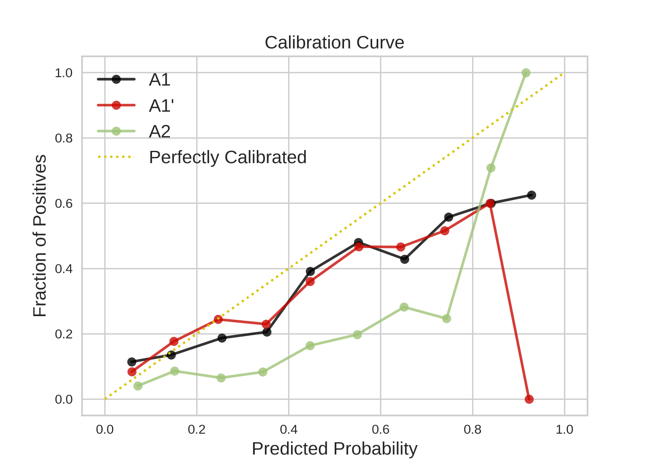 | 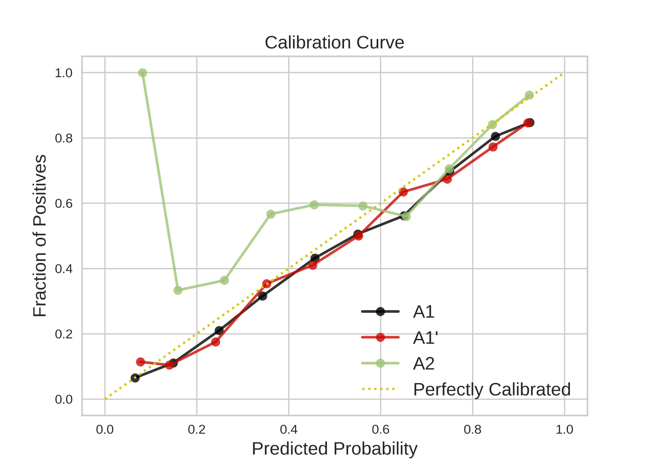 |
| 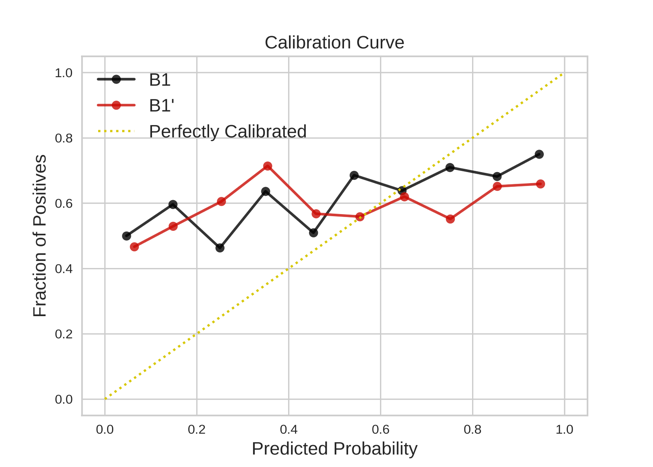 | 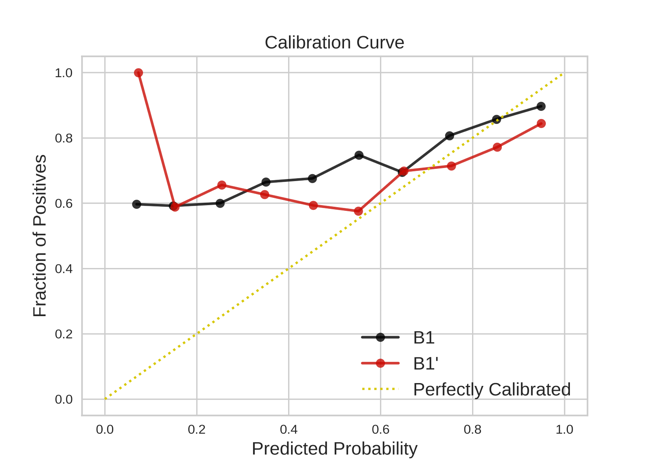 |
| 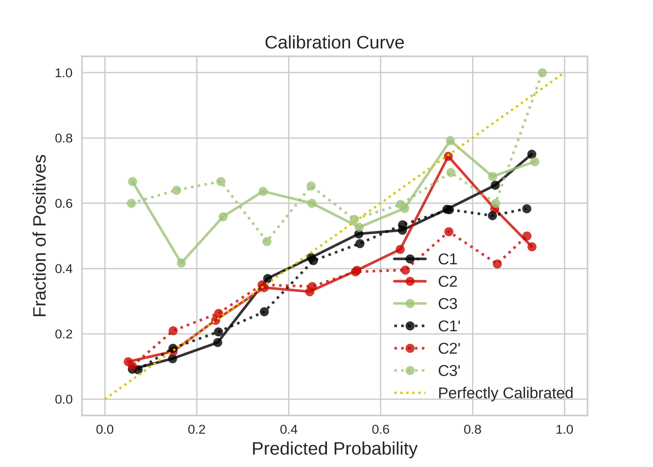 | 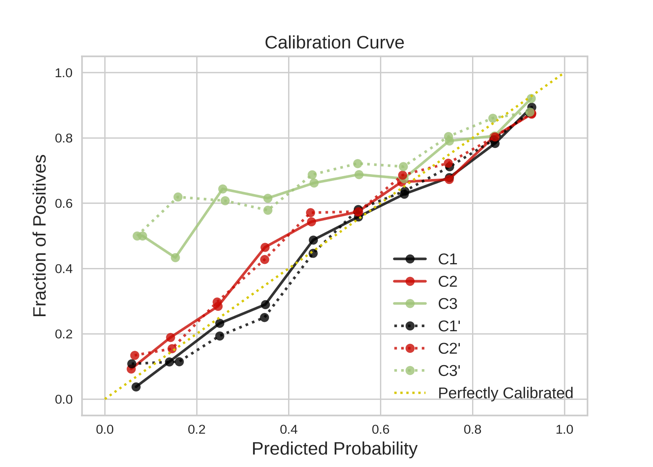 |

**Figure 7. Calibration curve of sub-group analysis of Septic vs. Non-septic patients using XGBoost.**

| **Horowitz index <162** | **Horowitz index ≥162** |
| --- | --- |
| 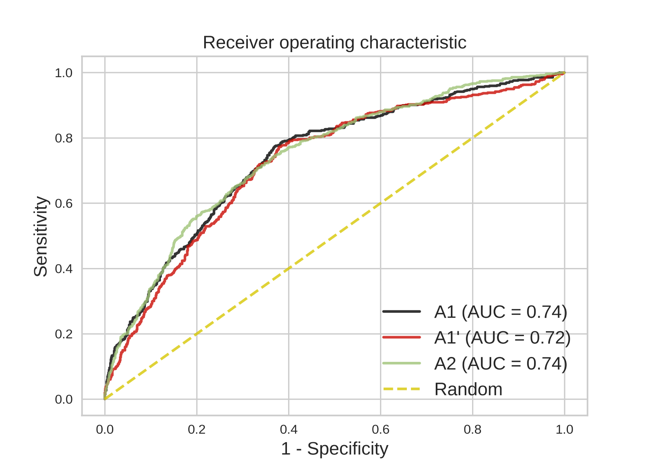 | 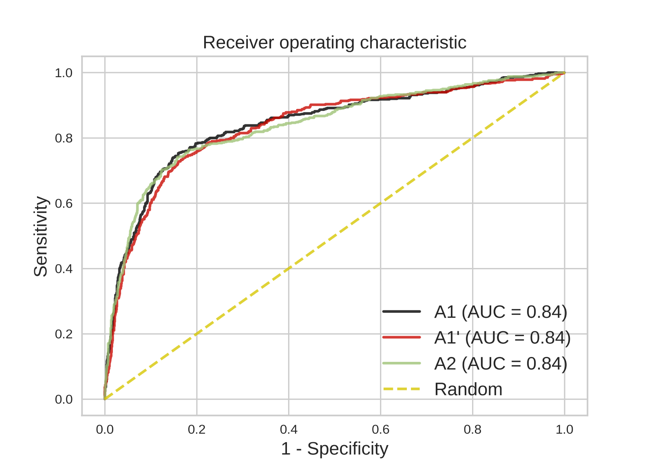 |
| 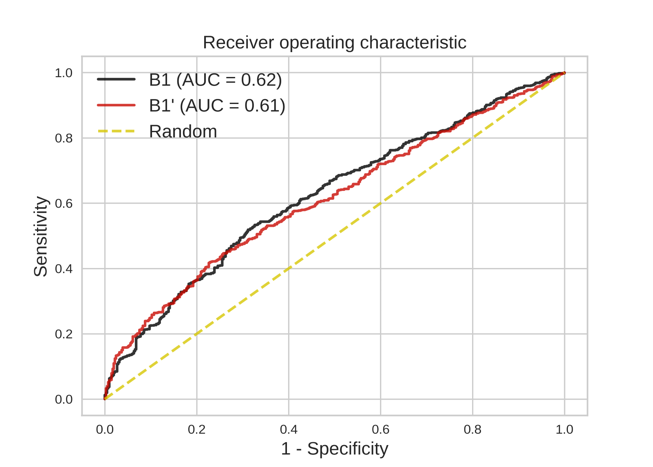 | 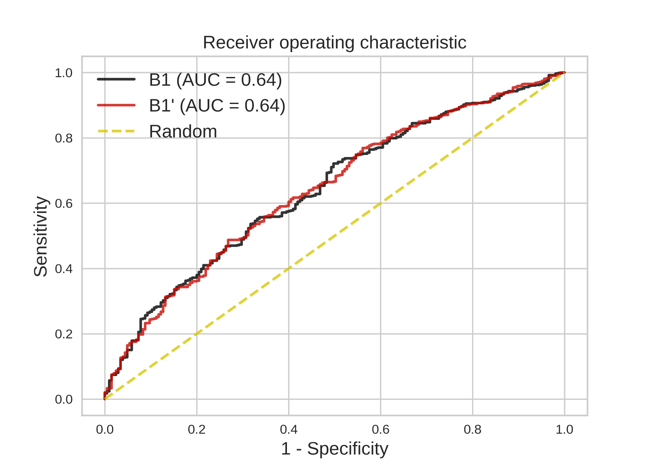 |
| 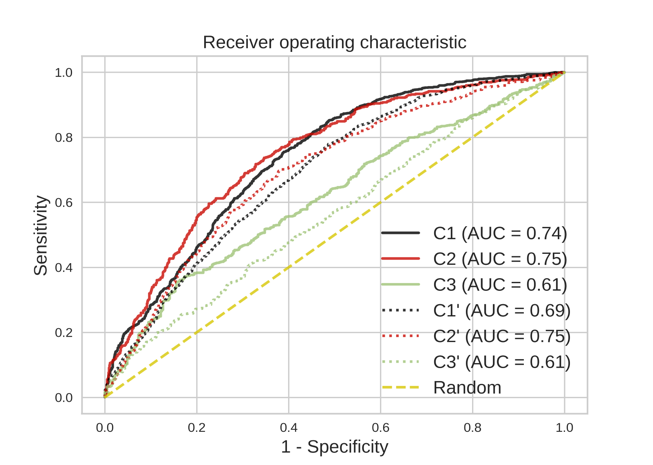 | 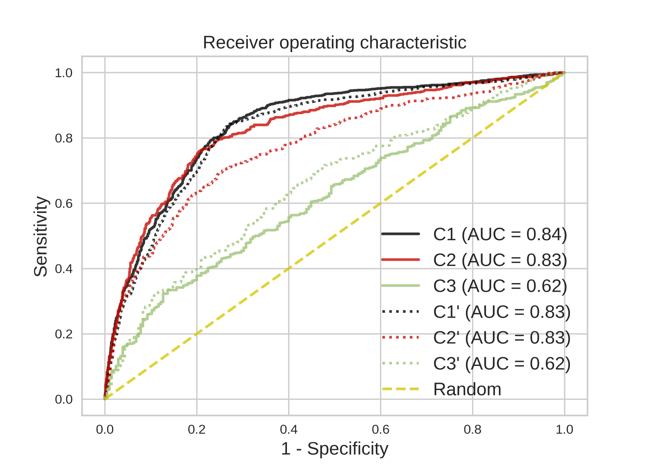 |

**Figure 8. AUROC of sub-group analysis based on Horowitz index using XGBoost.**

| **Horowitz index <162** | **Horowitz index ≥162** |
| --- | --- |
| 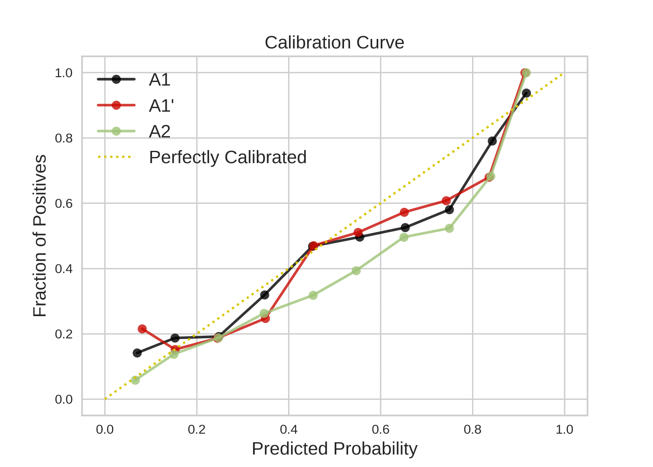 | 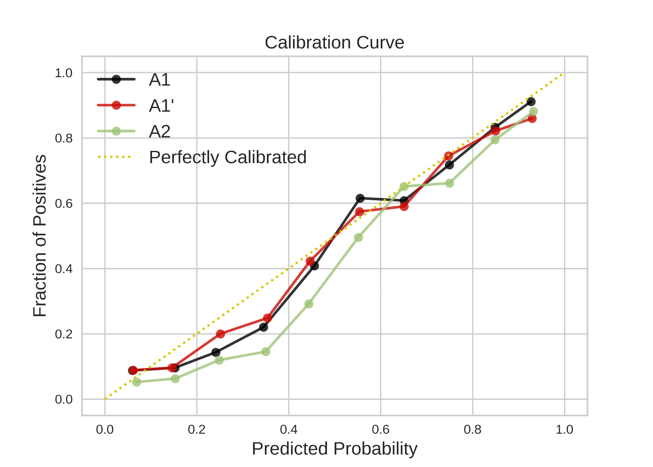 |
| 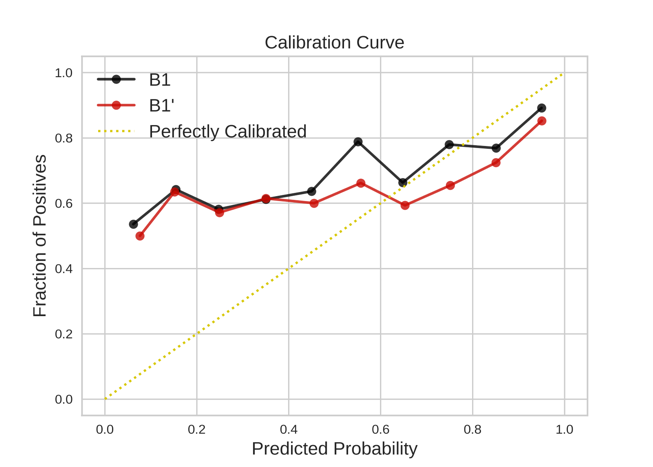 | 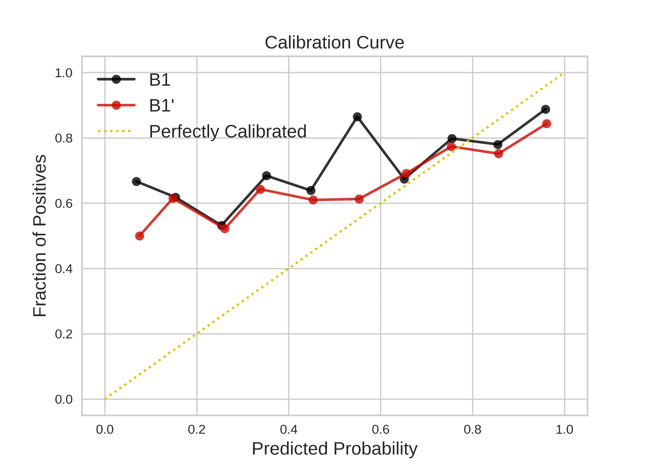 |
| 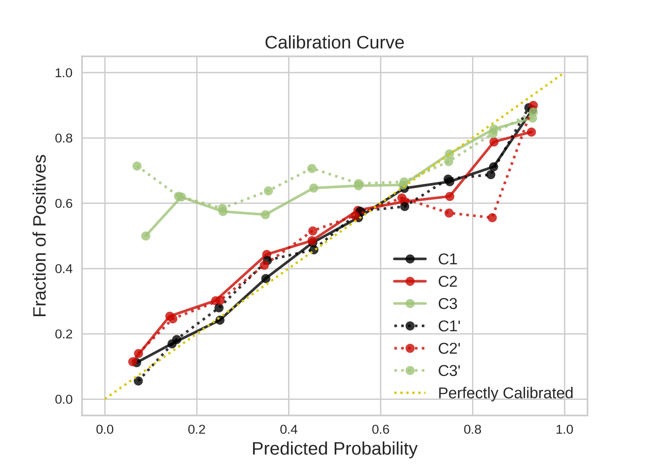 | 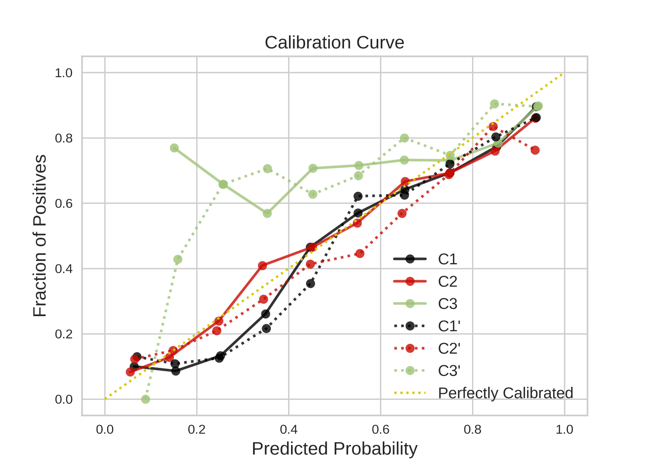 |

**Figure 9. Calibration curve of sub-group analysis based on Horowitz index**

**using XGBoost.**
